# Exploring the neuromagnetic signatures of cognitive decline from mild cognitive impairment to Alzheimer's disease dementia

**DOI:** 10.1016/j.ebiom.2025.105659

**Published:** 2025-03-27

**Authors:** Sinead Gaubert, Pilar Garces, Jörg Hipp, Ricardo Bruña, Maria Eugenia Lopéz, Fernando Maestu, Delshad Vaghari, Richard Henson, Claire Paquet, Denis-Alexander Engemann

**Affiliations:** a Université Paris Cité, Inserm UMRS 1144 Therapeutic Optimization in Neuropsychopharmacology, Paris, France; b Cognitive Neurology Center, GHU.Nord APHP Hôpital Lariboisière Fernand Widal, Paris, France; c Roche Pharma Research and Early Development, Neuroscience and Rare Diseases, Roche Innovation Center Basel, F. Hoffmann–La Roche Ltd., Basel, Switzerland; d Center for Cognitive and Computational Neuroscience, Universidad Complutense de Madrid, 28223, Madrid, Spain; e Department of Radiology, Rehabilitation and Physiotherapy, School of Medicine, Universidad Complutense de Madrid, Madrid, Spain; f Department of Experimental Psychology, Cognitive Processes and Speech Therapy, Universidad Complutense de Madrid, Madrid, Spain; g Department of Psychology, University of Cambridge, UK; h MRC Cognition and Brain Sciences Unit, University of Cambridge, CB2 7EF, UK; i Department of Psychiatry, University of Cambridge, UK

**Keywords:** Alzheimer's disease (AD), Mild cognitive impairment (MCI), Disease progression, Magnetoencephalography (MEG), Brain rhythms, Spectral power

## Abstract

**Background:**

Developing non-invasive and affordable biomarkers to detect Alzheimer's disease (AD) at a prodromal stage is essential, particularly in the context of new disease-modifying therapies. Mild cognitive impairment (MCI) is a critical stage preceding dementia, but not all patients with MCI will progress to AD. This study explores the potential of magnetoencephalography (MEG) to predict cognitive decline from MCI to AD dementia.

**Methods:**

We analysed resting-state MEG data from the BioFIND dataset including 117 patients with MCI among whom 64 developed AD dementia (AD progression), while 53 remained cognitively stable (stable MCI), using spectral analysis. Logistic regression models estimated the additive explanation of selected clinical, MEG, and MRI variables for AD progression risk. We then built a high-dimensional classification model to combine all modalities and variables of interest.

**Findings:**

MEG 16–38Hz spectral power, particularly over parieto-occipital magnetometers, was significantly reduced in the AD progression group. In logistic regression models, decreased MEG 16–38Hz spectral power and reduced hippocampal volume/total grey matter ratio on MRI were independently linked to higher AD progression risk. The data-driven classification model confirmed, among other factors, the complementary information of MEG covariance (AUC = 0.74, SD = 0.13) and MRI cortical volumes (AUC = 0.77, SD = 0.14) to predict AD progression. Combining all inputs led to markedly improved classification scores (AUC = 0.81, SD = 0.12).

**Interpretation:**

These findings highlight the potential of spectral power and covariance as robust non-invasive electrophysiological biomarkers to predict AD progression, complementing other diagnostic measures, including cognitive scores and MRI.

**Funding:**

This work was supported by: 10.13039/501100002915Fondation pour la Recherche Médicale (grant FDM202106013579).

**Research in context**

**Evidence before this study**

Alzheimer's disease (AD) is the most common cause of dementia, accounting for 60%–80% of cases. Mild cognitive impairment (MCI) is a critical stage preceding dementia, but not all patients with MCI will progress to AD. Non-invasive electrophysiology is a promising technique to improve the early identification of AD and assess the risk of cognitive decline. We reviewed existing research on using electroencephalography (EEG) or magnetoencephalography (MEG) recordings to predict the progression from MCI to AD dementia. Previous studies have shown that individuals with MCI or AD have slower brain rhythms, reduced complexity in brain signals, and decreased synchronisation between brain regions. These differences are attributed to neurodegeneration and impaired brain networks. While there is significant research using EEG recordings for the diagnosis of MCI and AD, fewer studies have explored their potential to predict cognitive decline and progression from MCI to AD dementia and even fewer have used MEG for this purpose. Importantly, methodologies and choice of spectral analysis measures differ across and within studies, potentially limiting comparability of results by inducing methods-related variance.

**Added value of this study**

This study applied a coherent spectral analysis methodology based on complex Morlet wavelets to evaluate MEG data from patients with MCI and model AD progression. This allowed us to study and compare different spectral metrics (power, power envelopes, phase interactions) and advanced statistical methods (Riemannian Geometry for processing interaction measures without source imaging) on an equal footing. Our study highlights the potential of MEG spectral power from 16Hz to 38Hz as a robust, brain-activity biomarker candidate to predict cognitive decline and AD progression, adding to conventional metrics like the Mini Mental state Examination (MMSE) score and structural MRI brain measures. Furthermore, Riemannian-based methods provided complementary insights into frequency-specific alterations of brain activity not detected by naive sensor space power, phase interactions and power envelope correlations.

**Implications of all the available evidence**

These findings add significant value to the existing evidence by demonstrating the potential of spectral power and advanced electrophysiological analytical techniques as non-invasive brain-activity biomarkers for AD progression, which could complement classical diagnostic measures and enhance early detection strategies. Our results hold promise for the development of screening strategies in large populations of individuals with MCI and align with the emergence of new AD disease-modifying treatments. Future research should focus on validating these findings in EEG studies and within the context of proteinopathies, going towards clinical application, and also expand the scope of our work to characterise neurodegenerative diseases other than AD.

## Introduction

Alzheimer's disease (AD) is the most common cause of dementia, accounting for an estimated 60%–80% of cases.^1^ Dementia is preceded by the mild cognitive impairment (MCI) condition, which is characterised by objective cognitive impairment in one or more domains with preserved functional independence.^2^ Not all patients with MCI will progress to AD dementia, as some can stay cognitively stable or even revert to normal cognition.^3^ Recently, new AD treatments have been developed with promising results.^4^^,^^5^ Biomarkers that are able to identify patients at a prodromal stage of AD are becoming essential, as treatments have shown to be more effective if given at an early stage. Biomarkers of AD progression are also needed to monitor the response to new treatments. However, currently established AD biomarkers for clinical use either require an invasive procedure (cerebrospinal fluid analysis), or are associated with high costs and limited availability, such as amyloid positron emission tomography (PET) and tau-PET scans, so they cannot be applied to a large populationsample worldwide, especially with repeated measures. Although novel fluid biomarkers, such as plasma amyloid-beta and phosphorylated tau, show significant promise and are increasingly used in large clinical trials, their integration into routine clinical practice requires further standardisation and validation for widespread application. Moreover, AD biomarkers (beta-amyloid protein, tau and phosphorylated tau that can be measured in cerebrospinal fluid, plasma or PET) have a relatively low sensitivity to synaptic dysfunction and may be insufficient to monitor modifications of brain function under treatment.^6^, ^7^, ^8^, ^9^ Furthermore, existing biomarkers do not account for the disjunction between the degree of brain pathology and its clinical manifestations, which refers to the concept of cognitive reserve.^10^ It has been shown that individuals with similar brain pathology can demonstrate differences in cognitive performance, probably underpinned by variations in functional network efficiency.^11^, ^12^, ^13^ This highlights an unmet need for brain activity-based biomarkers.

Non-invasive electrophysiology, such as electroencephalography (EEG) and magnetoencephalography (MEG), are promising techniques that could be complementary to other biomarkers currently in development for AD, including biological and imaging biomarkers.^14^, ^15^, ^16^, ^17^ Electrophysiology allows for the examination of neuronal activity across spatial and temporal scales, providing a window onto neuronal activity underlying cognitive functioning with high sensitivity to synaptic function.^18^^,^^19^ Information can be decoded from M/EEG by analysing the spatial and spectral organisation of brain activity using advanced statistical methods including machine learning.^20^, ^21^, ^22^ EEG and MEG have differential sensitivity to different configurations of neural activity (e.g. in terms of the orientation and depth of the dendritic currents that cause the electromagnetic field changes, and the effects of volume conduction and skull/scalp conductivities). While EEG is more suitable for clinical deployment due to better standardisation, scalability and costs, MEG offers better spatial resolution and often higher signal-to-noise ratio, making it a useful tool for research-grade discovery contexts.^23^^,^^24^ Moreover, recent developments in MEG sensors, like optically-pumped magnetometers,^25^ promise better scalability and reduced cost, i.e., more practical applications in the clinic. Despite some intrinsic differences between MEG and EEG, under favourable circumstances (e.g. spectral pattern of limited spatial complexity), MEG signatures could also be validated and adapted for biomarker studies using EEG.^26^, ^27^, ^28^ Although MEG is still undervalued in AD, it has the potential to significantly contribute to our understanding of how neurodegenerative diseases impact brain function, and could help predict future cognitive decline.^29^^,^^30^

A solid body of evidence exists that characterises temporal/spectral differences in resting-state brain activity between healthy controls and patients with MCI or AD dementia, as assessed with EEG. As reviewed by Cassani et al. (2018),^31^ compared to healthy controls, patients with AD or MCI usually show: 1) slowing of oscillatory brain activity, which is thought to result from loss of cholinergic innervation; 2) reduced signal complexity, which could be linked to neurodegeneration and fewer cortical connections; and 3) reduced synchrony, which likely reflects impaired communication of neural networks. While previous studies have used EEG/MEG in the context of MCI and AD dementia versus controls, fewer studies have used EEG to model progression from MCI to AD dementia^14^^,^^32^, ^33^, ^34^, ^35^, ^36^, ^37^, ^38^, ^39^, ^40^, ^41^, ^42^, ^43^, ^44^ and even fewer have used MEG for this purpose.^45^, ^46^, ^47^ Moreover, previous work has mostly focused on specific features (e.g. power in specific frequency bands), which also differ between studies, rendering comparisons difficult. Furthermore, pathology and medical treatments may alter neural dynamics at frequencies that are not well represented by standard frequency bands.^48^ This motivates approaches that analyse the frequency spectrum continuously,^49^^,^^50^ particularly as different pathological conditions can modulate electrophysiological signals at different spatial scales. For example, oscillatory activity in the alpha band (∼10Hz) tends to be maximal over posterior visual cortices, while that in the beta band (∼20Hz) tends to be maximal over motor cortex.^51^ In addition to changes in power, there can be changes in phase-coupling mechanisms^52^^,^^53^ and slow fluctuations of amplitude envelopes,^50^^,^^54^ as part of large-scale cortical network dynamics.^55^

This leads to the following two scientific questions for the present study: 1) can brain activity recorded with MEG help model progression from MCI to AD dementia? and 2) what features of the MEG signal are most characteristic of future cognitive decline and do they add information independently from anatomical MRI? To address these questions, we analysed neuromagnetic recordings from 117 patients with MCI, of which 64 later progressed to AD dementia (AD progression) while 53 remained cognitively stable (stable MCI) within 9-year follow-up, using 306-channel whole head MEG. To avoid bias due to pre-specified frequency bands, we conducted continuous spectral analysis using Morlet wavelets, covering the frequency spectrum from 1Hz to 64Hz in fine-grained intervals. This allowed us to define a common signal representation for various spectral measures used in the literature, i.e., power, covariance, and synchronisation measures including phase interactions, and power envelopes. As fine-grained regional changes in cortical activity might be lost when averaging across sensors, we employed multivariate analysis using the mathematical framework of Riemannian manifolds. These tools are well suited for capturing fine-grained spatio-spectral patterns of brain activity that are confounded by volume conduction and field spread—bypassing the need for source localisation with a biophysical model.^56^^,^^57^

## Methods

### Participants

We analysed MEG recordings from the BioFIND dataset^58^ comprising 158 clinically diagnosed participants with MCI according to Albert et al. (2011) criteria,^59^ recruited from two sites: 68 patients from the MRC Cognition & Brain Sciences Unit (CBU) at the University of Cambridge and 90 patients from the Laboratory of Cognitive and Computational Neuroscience at the Centre for Biomedical Technology (CTB), Madrid. Regarding participant inclusion and exclusion criteria, both sites followed the criteria established by Albert and colleagues^59^ to define MCI, even if there was a slight variation in their application. In Cambridge, participants were required to present an objective deficit specifically in memory domain tests, whereas in Madrid, the diagnosis required objective impairment in either the memory domain or other cognitive functions. A summary of the inclusion and exclusion criteria for both sites is provided in Table 1.

**Table 1:** Summary of the participants' inclusion and exclusion criteria for Cambridge and Madrid sites.

Criteria	Cambridge (CBU) site	Madrid (CTB) site
Diagnostic criteria	Albert et al. (2011)	Albert et al. (2011)
Objective cognitive impairment	Required in memory domain	Required in memory and/or other cognitive functions
Imaging	Brain MRI (or CT-scan if MRI contraindicated) to exclude other pathologies (vascular, tumoral …) and identify features consistent with MCI or AD pathology	Brain MRI to rule out a vascular disorder or any other type of neurological disease (e.g. tumor, infection …)
Functional independence	Required	Required
Psychiatric/neurological exclusion	No major psychiatric disorders	No psychiatric/neurological disorders, severe head injury, alcohol abuse, or medication that affects MEG
Biomarker usage	PET/fluid biomarkers were not used as standard	For some patients, biomarker evidence (APOE ε4, atrophy on MRI)

### Ethics

This study performed a reanalysis of anonymised data shared via controlled access to the BioFIND^58^ database via the Dementia Platform UK (DPUK) server platform. This secondary data analysis does not meet the definition of research requiring oversight by an institutional review board (IRB). We refer to the ethics information shared by the reference publication^58^: “The participants were pooled over a number of different projects, each approved by local Ethics Committees and following the 1991 Declaration of Helsinki. Participants consented to the collection and sharing of de-identified data for research purposes.”

We used a subset of 117 of the patients with MCI in BioFIND who had follow-up data. Of these, 64 subsequently progressed to probable AD dementia based on clinical criteria according to McKhann et al. (2011)^60^ (AD progression group), whereas 53 remained stable (stable MCI). For additional details, see the BioFIND dataset publication.^58^

Precise follow-up times were not documented on a patient-by-patient basis. According to the clinical scientists involved in data collection for this study, the follow-up period spanned approximately five years for the Madrid site and three years for the Cambridge site, although follow-up data for some participants could not be maintained throughout this period. During these follow-ups, participants who did not show progression were considered to have stable MCI. The follow-up duration is not available for some patients in the BioFIND dataset.

### Socio-demographic characteristics

There were no statistically significant differences for AD progression group and stable MCI for age or sex. However, we observed pronounced differences in years of education, though the direction of association actually suggested longer education in the AD progression group, suggesting that progression did not simply reflect worse education (Table 2). The Mini Mental Status Examination (MMSE) score was significantly lower in patients showing AD progression compared to stable MCI (p = 0.001) and therefore calls for statistical controls to rule out that potential differences in brain signals between AD-progression and stable MCI do not merely reflect baseline differences in cognitive function and progression.

**Table 2:** Means (and standard deviations) of socio-demographic characteristics of AD progression group and stable MCI.

Data characteristic	AD progression (n = 64)	Stable MCI (n = 53)	T/χ2 and p-value
Sex (M/F)	32/32	20/33	χ2 = 1.30, p = 0.253
Age (years)	73.03 (6.98)	72.47 (5.32)	T = 0.48, p = 0.632
Education (years)	10.24 (4.54)	8.58 (4.50)	T = 1.98, p = 0.05
Baseline MMSE	25.54 (2.82), range 17.0–30.0	27.15 (2.41), range 22.0–30.0	T = −3.28, p = 0.001

M: Male; F: Female; MMSE: Mini Mental State Examination.

In the course of this work, we discovered substantial differences in terms of socio-demographic characteristics between sites regarding the number of patients having progressed to AD versus stable MCI (Table 3). AD-progressors were overrepresented in the CBU site as compared to the CTB site. Moreover, sex, age, and education were markedly different between the sites. This poses certain challenges for the analyses developed in this study, as the unequal distribution of progression cases across sites has as a consequence that direct controlling for site-effects may reduce the signal-to-noise for statistical analysis modelling of progression risk as site is, to a certain extent, collinear with progression risk. We therefore carefully explored how controlling for site impacted results and paid close attention to the demographic characteristics as we built and evaluated models of AD-progression.

**Table 3:** Means (and standard deviations) of socio-demographic characteristics of patients by Madrid (CTB) and Cambridge (CBU) sites.

Data characteristic	CTB (n = 90)	CBU (n = 27)	T/χ2 and p-value
AD progression (Yes/No)	Yes: 41, No: 49	Yes: 23, No: 4	χ2 = 11.61, p = 0.001
Sex (M/F)	35/55	17/10	χ2 = 3.95, p = 0.047
Age (years)	73.99 (4.87)	68.74 (8.48)	t = 4.07, p = 0.0001
Education (years)	8.43 (4.47)	13.02 (2.88)	t = −5.02, p < 0.0001
Baseline MMSE	26.44 (2.64)	25.70 (3.10)	t = 1.22, p = 0.224

M: Male; F: Female; MMSE: Mini Mental State Examination.

### MEG data acquisition

MEG recordings were collected continuously at 1 kHz sample rate using an Elekta Neuromag Vectorview 306 MEG system (Helsinki, FI) at both CBU and CTB sites. MEG systems and scanning parameters were identical between sites, including continuous head position tracking and seating position of participants. Resting-state MEG data were recorded while participants were seated comfortably inside a magnetically shielded room and were asked to keep their eyes closed but not fall asleep. To minimize the risk of drowsiness during MEG acquisition, other measures were implemented, including continuous monitoring via CCTV. Movement parameters were also closely monitored, and MaxFilter was used to manage head movements exceeding 25 mm, although no significant movement was observed, indicating participants remained alert.

### Preprocessing

We analysed the first 2 min of resting-state eyes-closed MEG data for each patient. Data were processed in Python using the MNE software version 1.2.0.^61^ We decided to set the duration to the first 2 min to match the minimum across all participants. We believe it would be inadequate to use different durations for different individuals, as brain states can change over time, potentially introducing variability among participants. Moreover, using the first 2 min has the main advantage of minimising the likelihood of capturing drowsiness. The preprocessing steps were the following: MaxFiltering (SSS) was first applied to raw data to remove noise potentially arising from head movements and environmental noise. We applied the MaxFilter process using site-specific calibration and cross-talk correction files, as used in the study by Vaghari et al. (2022).^58^ Temporal Signal Space Separation (tSSS) was employed with an st_duration parameter of 10 s to enhance the separation of brain and external signals. The head origin was automatically determined (mf_head_origin = ‘auto’), and the destination was set to ‘dev_head_t’, ensuring consistent spatial alignment despite potential head movements. Data was resampled to a rate of 250Hz, after which a 0.5Hz–100Hz 4th-order Butterworth bandpass filter and 50Hz Notch filter were applied. To remove ocular and cardiac artifacts, spatial filtering was employed using the *signal space projection* (SSP) technique.^62^ The data were then cut in 10-s epochs and the *autoreject* algorithm was used to exclude noisy epochs.^63^

### Computation of MEG features

We focused on magnetometers as after MaxFilter cleaning, the information of gradiometers and magnetometers is merged and duplicated across both sensor types. Indeed, previous work has shown that after MaxFilter cleaning, spectral results obtained from gradiometers and magnetometers are highly similar^64^ and comparison of the power spectra between gradiometers and magnetometers on this dataset led us to the same conclusion. This facilitated data analysis through simpler handling and shorter computation times.

We computed spectral features with Morlet wavelets^50^^,^^65^ using the meeglet library.^66^ This wavelet approach implements Morlet wavelets spaced on a base-2 logarithmic grid such that the spacing between wavelets and their spectral smoothness increase log-linearly with frequency.^50^^,^^66^ Such wavelets are well suited for capturing the log-dynamic frequency scaling of brain activity^67^ and have proven useful in multiple EEG-biomarker applications.^49^^,^^68^^,^^69^ Moreover, Bomatter and colleagues^66^ found that such wavelets could outperform classical frequency-band definitions or linearly spaced power spectra on machine learning tasks. Another advantage of this approach is that across various spectral measures, the same spectral analysis method is used in this work, reducing methods-induced variance. The frequency of interest ranged from 1Hz to 64Hz, with a spectral smoothing (bandwidth) of 0.35 octaves and a spectral sampling of 0.05 octaves, yielding 121 wavelets. The bandwidth was chosen based on visual inspection of the average power spectrum across participants to control the trade-off between smoothness and spectral resolution. We applied log-frequency integration over Hz (common approach) rather than octaves (meeglet software default) to preserve 1/f characteristics as inspecting those might be insightful.

The following spectral features were computed: power spectral density, covariance estimated from the wavelet-convoluted time series, debiased squared weighted phase-lag index (dwPLI) and power envelope correlation (log of rectified wavelet-convoluted time series). This set of spectral features can capture distinct aspects of neural activity and are conceptually complementary. Moreover, previous EEG work has shown that spectral power, dwPLI and power envelope correlation revealed complementary facets of brain function in Huntington's Disease and pharmacological treatments thereof,^49^ hence, demonstrating the interest in empirically studying these metrics in the context of neurodegeneration.

#### Spectral power

Power-spectral density quantifies frequency-specific brain activity and is one of the most frequently visited EEG metrics. Power estimated from Morlet wavelets has proven useful in a number of biomarker applications including phenotyping and pharmacodynamic modelling.^49^^,^^68^, ^69^, ^70^, ^71^

#### Covariance

The covariance between sensors at a given frequency provides an extension of spectral power as it includes the power spectrum (diagonal term of covariance) but also provides information about the correlation between sensors (cross terms). This can help unmix hidden activity patterns and assess interdependence of neural signals: covariance-based modelling using Riemannian geometry^72^^,^^73^ has recently been explored in machine learning for biomarker applications to uncover brain activity without explicit MRI-based source localisation^56^ and proven useful on a number of prediction tasks.^66^^,^^74^ As a mathematical object, the covariance lives on the manifold of symmetric positive definite (SPD) matrices (its eigenvalues are strictly positive if no rank reduction is applied and sufficient data is available for estimation). Riemannian geometry enables computing symmetrical distances between covariances and provides tangent spaces with Euclidean distances, hence permitting the use of linear methods after tangent-space projection. Moreover, the affine invariant metric commonly used in Riemannian modelling has the interesting property that it defines a distance that is invariant to any affine transformation done to the covariance, which, in the context can arise from a difference in head position or artifacts. This offers an explanation for the observed robustness and efficacy of Riemannian models for MEG and EEG applications.^56^^,^^66^

#### Power-envelope correlation

This metric can detect non-instantaneous power correlations–regardless of their sign—which has been used to study synchronised signal amplitude changes between distant brain regions.^50^ The power envelope was defined as the log of the rectified wavelet-convoluted signal.^50^^,^^66^ Unless orthogonalisation is used,^50^ the power-envelope correlation matrix is also an SPD matrix, hence, amenable to Riemannian analysis.

#### dwPLI

The dwPLI metric captures changes in phase-synchronisation with reduced sensitivity to uncorrelated noise sources and increased statistical power to detect changes in phase-synchronisation compared to PLI.^53^ The dwPLI matrix is not an SPD matrix, hence, not amenable to Riemannian analysis without additional processing steps.

Together, these metrics bear the potential to provide complementary information on brain activity in different frequency ranges related to AD progression risk. We were in particular interested in studying metrics that can be defined in sensor space to assess their potential as biomarkers in clinical settings where an MRI may not be available (for accurate head modelling needed for MEG/EEG source modelling) and EEG is more commonly used than MEG. In this work, we therefore refrained from an interpretation of the power-envelope and dwPLI metrics in terms of functional connectivity, which, for proper interpretation, requires anatomical source modelling.

### MRI features

To assess the complementarity of MEG signals to anatomical information, we analysed T1-weighted structural MRIs from the BioFIND dataset. T1-weighted MRIs were processed using FreeSurfer version 7.3.2 software^75^ to compute global brain volumetric measures using the Desikan-Killiany atlas.^76^ We used all 64 regions provided by the atlas in our analysis, including the ratio of mean hippocampal volume to total grey matter based on the previous AD literature.^77^^,^^78^ Reduced hippocampal volume is a well-established marker for AD^6^ where its normalisation relative to total grey matter volume helps to account for individual differences in total cortical volume.

### Statistics

#### Missing values

For comparison of socio-demographic characteristics we identified 15 missing values for education and 5 missing values for MMSE. To facilitate exploration, we applied simple mean imputation independently for each variable, using the mean value calculated over all subjects. Given the smaller number of subjects and analyses concerned, this provided a reasonable tradeoff between facilitating exploratory analyses and statistical accuracy. MRI was not available for 13 participants. As the MRI was entirely absent, we refrained from imputation, leading to a reduced subset of participants for the comparisons between logistic regression models described below.

Recognising the limitations of mean imputation given its capacity for distorting statistical estimates, we employed an iterative imputer (predicting missing values from other variables) in our final classification model in which we included all variables of interest (see section *data-driven classification model* below). This approach ensured that no participant was excluded in the final model.

In the following, we define a number of statistical analyses. Some of those involve methods specifically developed within the field of neuroimaging or involve custom metrics based on resampling methods which may not be known by name in the wider biomedical literature. Where appropriate, we will introduce a nomenclature *T*_*<number>*_ to facilitate referencing and reporting.

#### Uncorrected visual inference by frequency [T_1_]

For spectral power analysis, we computed and plotted the average log power spectra over all sensors between 1Hz and 64Hz. We used non-parametric bootstrap resampling to obtain confidence intervals and permutation tests of the mean difference to obtain (uncorrected) p-values for the plotted average difference between groups along the frequency spectrum. Both were implemented using SciPy's^79^ bootstrap and permutation_test functions, respectively, with 9999 iterations (default).

#### Clustering permutation-testing across frequencies [T_2_]

To correct for multiple comparisons and take into account the correlation between frequencies, we used clustering-permutation tests along the frequency spectrum (permutation F-test) as implemented in MNE Python.^61^ The permutation F-test was used to compare average spectral power between groups at frequencies ranging from 1 to 64Hz. For power envelope correlation and dwPLI, we computed the average metrics averaging cross-terms over the rows and columns of all sensors, related to the mean degree from graph theory. A permutation F-test was used to compare average metrics between groups at frequencies ranging from 1Hz to 64Hz, using an F-test at each frequency followed by a cluster-permutation statistic. To reduce the dependency of the results on the particular choice of cluster-inclusion values, we used Threshold-Free Cluster Enhancement (TFCE) as implemented by the MNE-Python software with 10000 iterations.^80^ This procedure intrinsically controls for multiple comparisons.

#### Clustering permutation-testing across frequencies and sensors [T_3_]

We analysed each metric of interest for every sensor and each group. For power-envelope correlations and dwPLI, we computed the average metric aggregating per sensor cross-terms over the rows and columns. To test for topographic differences, we computed spatio-spectral permutation tests adapted from MNE-Pythons spatio-temporal permutation testing procedures (replacing time by frequency). To reduce the dependency of the results on the particular choice of cluster-inclusion values, we used TFCE as implemented by the MNE-Python software with 10000 iterations.^80^ This procedure intrinsically controls for multiple comparisons.

#### Riemannian distance MANOVA [T_4_]

As a complementary method for using spatial information in statistical analysis, we explored multivariate Riemannian distance MANOVA F-test^81^ conducting non-parametric distance MANOVA to test for group differences as implemented in the PyRiemann library.^82^ In the first phase of this work, we applied the Riemannian MANOVA to the covariance features for which we had the expectation that this approach might uncover additional signals compared to power spectral analysis by using multivariate distances rather than univariate tests and considering not only the power (diagonal term of covariance) but also the instantaneous correlations between sensors (cross-terms of covariance). As a distance, we used the Riemannian affine invariant distance which has been used with great success in different machine learning applications for MEG and EEG including brain computer interfaces, brain-age prediction and sex classification.^56^^,^^66^^,^^72^^,^^73^ Theoretical analysis and empirical benchmarks have shown that Riemannian metrics mitigate distortions due to electromagnetic field spread and provide latent representations well suited to statistically isolate information related to cortical current generators.^56^^,^^83^ Combined with the distance MANOVA,^81^ this procedure can be expected to provide a useful multivariate method for detecting group differences in brain activity. The affine-invariant Riemannian metric expects covariances to be SPD, hence, to be full rank. Maxfiltering projects noise components from the data and commonly reduces the data rank to 65. To obtain valid SPD matrices, we used the method from prior work by Sabbagh and colleagues,^83^ which linearly projects covariance matrices to the smallest common subspace using principal component analysis. We, hence, projected the covariances to the rank of 65 and applied a regularization parameter of 10 × 1^−15^ and scaling of 1 using the coffeine library.^56^

Prior to this work, we have not yet explored the applicability of Riemannian methods to power-envelope correlations and the dwPLI metric. While power envelope correlations should behave similarly to covariance matrices and yield SPD matrices, this is not the case for dwPLI. In our efforts at supporting Riemannian analysis for these two metrics we found that the same settings that worked for covariances also worked for power envelope matrices. By contrast, for dwPLI, we used the nearest symmetric-positive-semidefinite matrix algorithm^84^ to obtain an SPD matrix which were previously used for SPD-manifold modelling with very noisy EEG data. Moreover, we had to use extreme low-rank regularisation (rank = 5 instead of 65) to obtain a useful SPD manifold for dwPLI.^85^

#### Logistic regression model of AD-progression risk

**Fig. 1:**
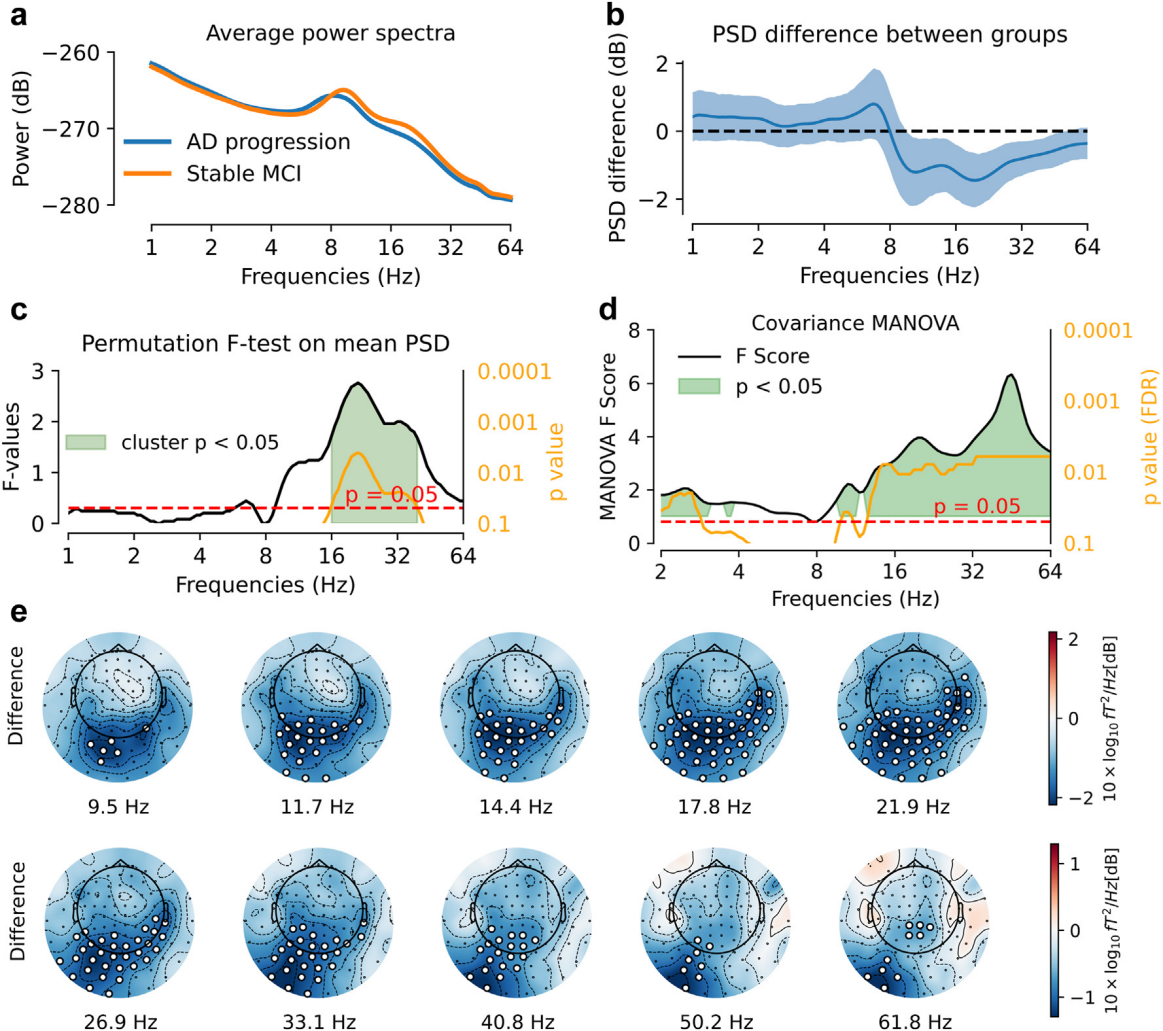
**AD progression was characterised by altered spectral power and covariance profiles.** Analysis was based on all n = 117 cases. (**a)** Power spectra averaged over all sensors: upon visual inspection, AD progression was associated with reduced spectral power at baseline in frequencies ranging from 10Hz to 40Hz. (**b)** Mean spectral power difference between groups (blue line) and 95% confidence interval computed by bootstrap (blue shaded area). (**c)** TFCE permutation F-test (two-tailed) on mean spectral power, showing significant power difference between AD progression and stable MCI at frequencies ranging from 16Hz to 39.4Hz [T_2_: p < 0.05]. The black line represents F values [T_2_], the orange line shows the TFCE p-value [T_2_]. The shaded green region indicates frequencies with T_2_: p < 0.05. (**d)** Comparison between groups based on covariance using Riemannian-distance MANOVA with dimensionality reduced to 65 components. The black line represents pseudo-F scores [T_4_], the orange line the p-value [T_4_] corrected using the False Discovery Rate (FDR). The shaded green region indicates frequencies with T_4_: p < 0.05. Specifically, significant differences were identified between 1Hz to 4Hz and 10Hz–64Hz (all p-values <0.05). After applying the FDR correction, significant differences remained for frequencies between 1Hz and 2.8Hz, 10.2Hz–10.9Hz, 12.6Hz–64Hz, as shown by the orange indicators. (**e)** Topographical maps of spectral power difference between groups, showing reduced spectral power over posterior sensors in AD progression group in frequencies from 9.5Hz to 61.8Hz. The white dots indicate significant differences [T_3_: p < 0.05]. For simplicity, here we only depict topographies at least including five significant sensors. This result has been obtained by TFCE spatio-temporal cluster permutation test (two-tailed).

To explore potentially additive explanations of AD progression risk we constructed a logistic regression model. This was motivated by the small sample size of the BioFind cohort. Traditional logistic regression analysis in biomedical applications (unlike machine learning) does not support shrinkage and focuses on explaining rather than predicting.^86^^,^^87^ To avoid overfitting, we followed Harrell's rule^88^ of selecting as a number of variables the number given by 10–20% of the samples in the smaller class, i.e., ⌊n=27×0.2⌋=5 where ⌊x⌋ is the floor function. We therefore limited the number of variables to 5. To control for variables that showed the strongest differences between sites or were known to be systematically linked to progression, we focused on age, education and MMSE as baselines. As site was potentially collinear with progression status (Table 3), which may pose particular issues for classical (non-regularised) logistic regression, we later on investigated the impact of controlling for site effects. We then sought to identify the most representative MEG variable and the most representative volumetric MRI variable. For MRI, hippocampus atrophy is a hallmark of AD. To account for individual differences in head size, we calculate the ratio of hippocampal volume to cortical volume. For MEG (and EEG), spectral power is one of the most prominently investigated electrophysiological features in the AD field. We therefore focused on log power but wished to base our precise feature definition on the exploration of spectral patterns investigated in this exploratory study. Our investigations highlighted activity over posterior sensors in the beta frequency range (cf. Fig. 1e), which we then reused for the logistic regression model (averaged over sensors and frequencies). Although we found value in exploring power-envelope connectivity and dwPLI metrics, these metrics were less discussed in the literature and we were uncertain about a compelling single-variable summary for these metrics suitable for the logistic regression. We hence revisited those features later in an exploratory data-driven model. An overview of the different variables used for logistic regression is presented in Table 4.

**Table 4:** Description of logistic regression model.

Model	Model definitions
Model 1	progression ∼ age + education + MMSE
Model 2	progression ∼ age + education + MMSE + Hippocampus/Total grey matter ratio (MRI)
Model 3	progression ∼ age + education + MMSE + cluster MEG power
Model 4	progression ∼ age + education + MMSE + cluster MEG power + Hippocampus/Total grey matter ratio (MRI)

All variables represented continuous or ordinal measurements. To ensure comparable numerical ranges, all variables were z-transformed using standard scaling.

We implemented logistic regression models using the R Software Version 2022.12.0 + 353. We took a step-wise model comparison approach using the difference of −2 in the ΔAIC (Akaike Information Criterion) as selection criterion to identify the most appropriate model in terms of trading expected generalisation error against model complexity, as recommended by Burnham and Anderson.^89^ We computed marginal effects to perform inference in terms of implied changes in probability (a nonlinear function), rather than the linear predictor of the logistic regression model which the model coefficients refer to.^90^ We relied on the average marginal effect (AME) which, for logistic regression, tracks the average change in probability of belonging to the reference class for a unit change in the input variable conditional on all other variables. Marginal effects were computed alongside classification metrics (area under the curve, sensitivity, specificity). To obtain a more nuanced interpretation of the models’ implied classification behaviour, we computed the 95% confidence intervals for the area under the curve (AUC) of the receiver operating characteristic (ROC) using bootstrap resampling with 2000 iterations. For each bootstrap sample, the dataset was resampled with replacement, and the model was re-fitted to obtain the predicted probabilities (nonlinear model output), followed by calculating the AUC. This approach provided a distribution of AUC values for each model, allowing us to estimate the 95% confidence intervals and assess the variability in model performance. For specific comparisons between pairs of models, we computed bootstrapped confidence intervals for the difference in AUC from the same procedure with fixed random seeds ensuring the comparability of bootstrap distributions.

Parameter inference in logistic regression is estimated simultaneously and parameters are conditionally dependent. Therefore, corrections for multiple comparisons are typically not performed.

#### Data-driven classification of AD progression and conditional permutation importance [T_5_]

The logistic regression approach can provide insights into the direction of the impact of value changes in one variable on the outcome, conditional on all other variables. Yet, it has the limitation that we used a small number of pre-selected variables, which forced us to make choices leading to neglecting information present in other variables. It also increased the risk of circularity as our choice of the MEG power was based on visual-statistical exploration. We therefore developed an alternative complementary modelling approach that applied data-driven machine learning methods to the problem of classification of AD progression and allowed us to include all variables of interest. We focused on machine learning methods which by design accommodate a large number of variables via regularisation e.g. parameter shrinkage. We incorporated all MEG metrics i.e., covariance (contains power on its diagonal terms), power envelope correlation, dwPLI, covered by the previous analyses, but importantly, for all sensors and all frequencies without pre-specified averaging or selection of individual features. To avoid redundant computation, we included every 4th frequency between 1Hz and 64Hz. Given the spectral smoothing implied by Morlet wavelets, this should be sufficient for the machine-learning methods applied here as previous work showed that, for a wide range of tasks, prediction performance tends to saturate at around 10 wavelets covering the frequency spectrum.^66^^,^^74^ We also included the entire set of volumetric outputs from FreeSurfer for the Desikan-Killiany atlas,^76^ leading to 64 values (for an overview of features, see Table 5).

**Table 5:** Description of the features included for the data-driven classification model (full model).

	Site	Age	Sex	Education	MMSE	MEG covariance	MEG power envelope correlation	MEG dwPLI	MRI volume
# inputs	1	1	1	1	1	102 × 102 x 31 = 322,524	102 × 102 x 31 = 322,524	102 × 102 x 31 = 322,524	64
# parameters (first layer)	1	1	1	1	1	65 x (65–1)/2 × 31 = 64′480	65 x (65–1)/2 × 31 = 64′480	102 x (102–1)/2 × 31 = 159,681	64
# parameters (second layer)	9								

Input variables for MEG represent symmetric matrices of 102 magnetometers at 31 frequencies. As Riemannian SPD manifolds require full-rank data and Maxwell filtering reduced the data rank of all 102 magnetometers and 204 gradiometers (after data fusion) to 65, rank reduction is applied for Riemannian models. Vectorization using the upper triangle follows the formula p×(p−1)/2 where p is the number of features, i.e., MEG channels in this case.

To find modality-specific classification rules while facilitating between-modality comparisons, we used the feature stacking approach consisting of a first-layer of modality-specific models and a second layer combining the cross-fitted predictions from the first layers. This allowed us to combine and compare separate high-dimensional and modality-specific classifiers with modality-specific regularisation on an equal footing as each output of the first-layer models is represented by a single variable in the second model.

As the sample size was small, we preferred linear methods trading bias for variance. For the first level, we used a fast regularized fast linear classifier (scikit-learn: RidgeClassifierCV) returning the linear decision function as in Bomatter (2024),^15^^,^^56^^,^^66^^,^^91^^,^^92^ which is similar to the linear sublayers in Chamma et al., 2024.^93^ In the second layer, we then used a regularised logistic regression model (scikit-learn: LogisticRegressionCV) providing calibrated probability outputs, thereby as in prior work, reserving nonlinear functions for the second layer.^15^^,^^93^ As regularization parameters, the first and second layer models used 50 values on a logarithmic grid between −3 and 5. As in Engemann et al., 2020,^15^ we employed a 10 x repeated 10-fold cross-validation (CV) scheme, leading to 100 CV splits in total. For every repetition, CV folds were propagated as groups for leave-one-group-out cross-validation in the second layer, which avoided sample leakage. CV splits were fixed and reused across all models to ensure strict comparability. To deal with missing values (MMSE, education, MRI), we used an iterative imputer (scikit-learn default of Bayesian ridge regression) inside the CV pipeline (fitted on training, applied on testing data) and standard scaling was used to enforce comparable value ranges. For MEG features, the same preprocessing was applied as described above for Riemannian-stance MANOVA. As dwPLI is not an SPD matrix, we vectorized using the upper triangle of the matrix, which led to better results than the low-rank (rank = 5) SPD manifold based on the nearest symmetric semi-positive definite algorithm used for dwPLI MANOVA above.

To analyse the potentially additive merit of the different input variables, we conducted a statistically controlled analysis of variable importance using the conditional permutation importance (CPI) method^93^^,^^94^ as implemented in the hidimstat package (https://mind-inria.github.io/hidimstat/api.html). CPI provides permutation-based assessment of variable importance while accounting for correlations and redundancies between input variables and therein overcomes the limitation of traditional permutation importance^95^^,^^96^ which was proven not to control type-1 error even if variables are correlated.^94^ Rather than marginally permuting variables and then computing the difference in loss, CPI estimates conditional importance by comparing the loss after predicting a variable from the other variables, permuting and re-adding its residuals—thereby isolating the information brought by a variable that is not shared with other variables. As the interpolation model for conditional inference, we used ridge regression (as the regression analogon of logistic regression) with the same set of regularization parameters. The log loss was used as the importance metric (default method for CPI with classification models in hidimstat). We used 500 permutations per CV split and computed a cross-fitted CPI statistic by dividing the mean CPI score across CV splits by its standard deviation across CV splits, yielding a pseudo t-statistic. As CV splits are not statistically independent, we applied Nadeau's & Bengios corrected t-test^97^ which takes into account the CV scheme for estimating appropriate degrees of freedom in the variance estimate. As in prior work,^74^^,^^94^^,^^95^ a one-sided test was used as one usually expects ML models to perform worse when removing predictors.

As CPI estimates were conditionally dependent and CPI intrinsically controls type-1 error, we did not apply correction for multiple comparisons. Of note, the choice of a nominal alpha level can seem particularly arbitrary in the context of a ML model as performance drops that are not statistically significant may still be economically or practically significant. We therefore also showed the CPI scores alongside uncertainty estimates and aimed for a nuanced description of results.

### Role of funders

D.E, P.G, & J.F.H. are full-time employees of F. Hoffmann-La Roche Ltd. (see *Declaration of Interests* for details). The employer of the authors did not have any role in this project, e.g. study design, data collection, data analyses, interpretation, or writing of the report. S.G. received a grant from the Fondation pour la Recherche Médicale (FRM) to support her PhD research (FDM202106013579). The FRM did not have any role in study design, data collection, data analyses, interpretation, or writing of the report.

## Results

### Analysis of frequency-dependent brain activity

AD progression was associated with reduced averaged spectral power compared to stable MCI in frequencies ranging from 16Hz to 40Hz [T_2_: p < 0.05] (Fig. 1a–c). An analysis based on the full covariance matrix using Riemannian-distance MANOVA F-test confirmed the high-frequency difference and, in addition, uncovered significant group differences in frequencies from 1Hz to 4Hz (Fig. 1d) while confirming the high-frequency effects [T_4_: p < 0.05]. Topographical analysis via spatio-spectral clustering statistics showed reduced spectral power over posterior sensors for AD progression in frequencies from 9.5Hz to 62Hz [T_3_: p < 0.05] (Fig. 1e).

As a first sensitivity analysis, we linearly adjusted the power spectra for MMSE. The significant group differences in spectral power remained, with the AD progression group showing reduced 16Hz–38Hz power over left parieto-occipital sensors [T_3_: p < 0.05] (Supplementary Figure S1). This analysis suggests that reduction in log power over posterior MEG sensors in the beta-band range between 16 and 38Hz was a characteristic feature of patients who later progressed to AD, highlighting a narrower spatio-spectral window after controlling for MMSE.

### Multimodal analysis of AD progression risk

**Fig. 2:**
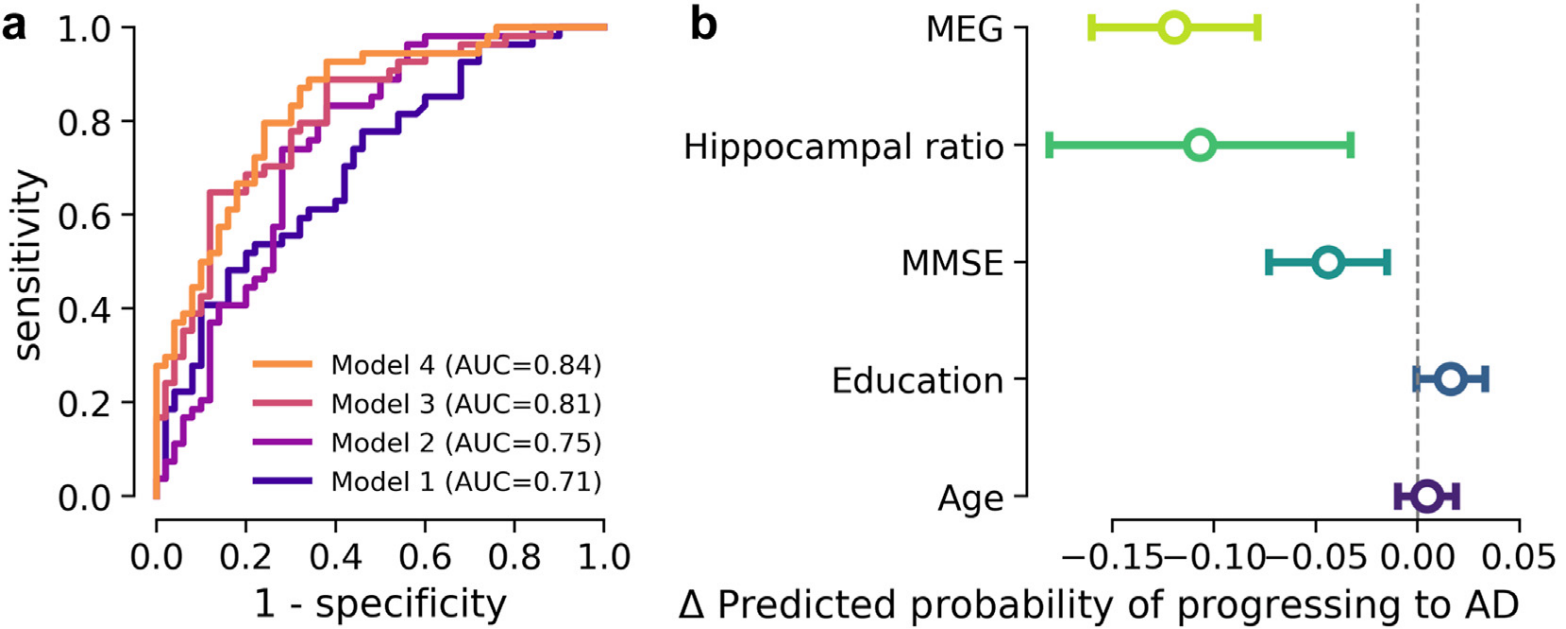
**MEG spectral power, MRI, and MMSE offered additive explanations of AD progression.** Analysis was based on the subset of n = 104 containing both MEG and MRI. **(a)** ROC curves of four logistic regression models to predict progression to AD dementia. **(b)** Marginal effects display of logistic regression model of risk of progressing to AD dementia using the following covariates**:** MEG 16–38Hz spectral power in parieto-occipital sensors, Hippocampus/Total grey matter ratio, MMSE, education and age. Higher values of MEG 16–38Hz spectral power in left parieto-occipital sensors, higher Hippocampus/Total grey matter ratio and higher MMSE were significantly associated with a reduced risk of progression to AD dementia conditional on all other variables. A higher level of education showed weaker effects in increasing the probability of progression to AD dementia.

This raises the question of whether this difference in brain activity overlapped with cognitive function and characteristic anatomical brain alterations. To explore additive explanations of progression risk, we constructed logistic regression models comparing MEG power against key demographic, clinical and MRI features (cf. Table 4). We sequentially explored four increasingly complex logistic regression models for the risk of AD progression (Fig. 2a) in terms of model fit and complexity (Aikake's information Criterion, AIC), average marginal effects and area under the curve (AUC) for implied discrimination behaviour. We first constructed a baseline with clinical and demographic variables only: Model 1 combined age, education and MMSE had a 0.71 AUC (95% CI: 0.62–0.82), a sensitivity of 65% and a specificity of 58%. In a next step, we constructed an enhanced baseline including MRI: Model 2 combining age, education, MMSE and hippocampus/total grey matter ratio had a 0.75 AUC (95% CI: 0.67–0.85), a sensitivity of 72% and a specificity of 72%. Then, we created a model that combined clinical and demographic variables with MEG to explore the added value of electrophysiology to clinical information: Model 3 combining age, education, MMSE and cluster MEG power better explained the data than Model 1 and 2, with an AUC of 0.81 (95% CI: 0.74–0.90), a sensitivity of 78% and a specificity of 70%. Thus, discrimination capacity increased from Model 1 to Model 2 and further improved markedly from Model 2 to Model 3. Finally, Model 4 combining age, education, MMSE, cluster MEG power and Hippocampus/Total grey matter ratio achieved slightly better results than Model 3 with an AUC of 0.84 (95% CI: 0.78–0.92), a sensitivity of 80% and a specificity of 74%.

As models were compared on the same data, the uncertainty in AUC estimates was coupled between pairs of models. We therefore explored the difference in AUC more systematically using bootstrap resampling. When comparing Model 3 and Model 4, the confidence interval for the difference in AUC ranged from −0.004 to 0.08, indicating a small possibility that there was no difference between the models. However, the majority of bootstrap replicas (93%) showed a difference greater than zero. In contrast, the comparison between Model 2 and Model 4 showed a much clearer, significant difference, with a confidence interval from 0.026 to 0.179, highlighting a substantial improvement in Model 4's discrimination capacity over Model 2 that only used MRI.

Importantly, the AIC values capturing the bigger picture of model fit versus complexity-driven risk for generalisation, showed that Model 4, with an AIC of 113.4, had best trade-off between fit and complexity, followed by Model 3 (AIC: 118.6), Model 2 (AIC: 132.6), and Model 1 (AIC: 136.9). Comparisons between models indicate a consistent benefit in including MEG and MRI features, with significant AIC reductions between the different models (AIC difference above −2 for all model comparisons), suggesting that both MRI and MEG can provide valuable information that could translate into improved prediction capacity.

Furthermore, Model 4 offered us the opportunity of inspecting the conditional relationship between hippocampal volumes and MEG power regarding progression risk (Fig. 2b). Inspecting the marginal effects, we found that higher hippocampus volume relative cortical volume as well as higher MEG power in the beta-frequency range lowered the risk of AD progression in a statistically independent fashion and conditional on MMSE scores, education and age. For both, MEG, and MRI the reduction of progression risk was on the order of 10% (AME = −0.12, z = −5.75, p < 0.001 and AME = −0.11, z = −2.83, p = 0.005, respectively), whereas MMSE accounted for a reduction around 5% whereas education seemed to increase the risk for AD (AME = −0.04, z = −2.99, p = 0.003 and AME = 0.02, z = 1.87, p = 0.06, respectively). This may seem curious but could be explained in terms of site effects as the general population accessed at the Cambridge site may have a higher education status compared to other cities.

### Sensitivity analyses: site effects and spatial averaging

*Site effects.* As the ratio AD progression to stable MCI was imbalanced across sites, site is a proxy for progression, hence, controlling for site can reduce statistical power for the analyses presented. We performed a sensitivity analysis to analyse robustness of results to site effect. Supplementary Figure S4 shows site-adjusted power spectra, averaged over all sensors. The overall pattern was preserved in the data pointing at reduction of power in the beta-band frequency range as shown by the bootstrapped differences of mean (Supplementary Figure S4b). However, controlling for multiple comparisons with cluster-permutation testing and keeping the previous detection thresholds, we found no statistically significant differences between groups after adjustment on site. However, we found a non-significant cluster between 18.4Hz and 21.9Hz at an alpha of [T_2_: p < 0.10] for AD progression versus stable MCI (maximum p-value of the cluster = [T_4_: 0.09]), again, preserving the overall pattern described above. Likewise, spatio-spectral cluster testing topographical maps showed a trend towards decreased spectral power from 14.4Hz to 25Hz in the posterior regions in the AD progression group using a p-value threshold below 0.10.

As logistic regression estimates a non-linear function of the input data, the impact of controlling for site may have different consequences. We have added site as a predictor to the previous Model 4 (reported in Fig. 2) and present its marginal effects in Supplementary Figure S5. It can be seen that despite the pronounced and expected site effect (given the strong imbalance of progression rates), the significantly additive contribution of MEG and hippocampal ratio to explaining progression risk was preserved.

*Spatial averaging.* We next performed a sensitivity analysis to assess the impact of spatial averaging over all sensors without using the cluster for multimodal analysis of AD progression risk. MEG 16–38Hz average spectral power over all sensors (adjusted for MMSE), hippocampal ratio and MMSE remained additive in the logistic regression model (Supplementary Figure S3b). Again, models including MEG showed stronger discrimination capacity than other models (Supplementary Figure S3a).

*Head alignment.* Finally, we performed an additional sensitivity analysis to assess the impact of head position alignment, using Maxfilter's “trans—default” option (Supplementary Figures S9 and S10). Conclusions were unchanged for the averaged spectral power differences regardless of whether the power spectrum was adjusted for MMSE or not. The spatial pattern changed, but remained significant before adjusting for MMSE, though not when regressing out MMSE. MEG spectral power, hippocampal ratio and MMSE remained additive in the logistic regression model when using the cluster without MMSE correction but controlling for MMSE in the model (Supplementary Table S1).

### Exploration of phase and amplitude interactions

**Fig. 3:**
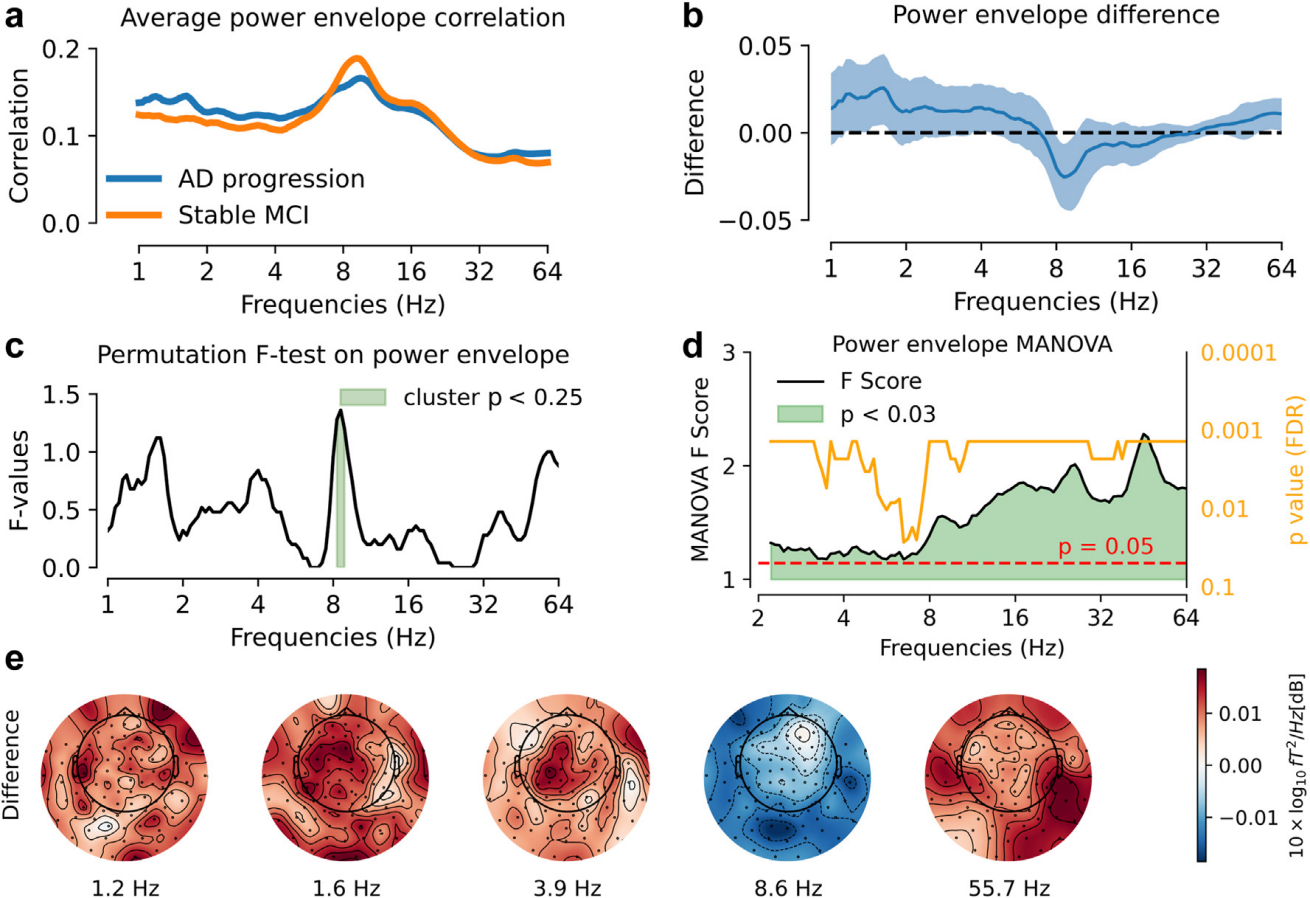
**AD-progression showed altered frequency-architecture in non-instantaneous power correlations.** Analysis was based on all n = 117 cases. **(a)** Average power-envelope correlation over all sensors. AD progression was visually associated with increased correlation below 4Hz and decreased correlation around 8Hz. **(b)** Power envelope correlation difference between groups (blue line) and 95% confidence interval computed by bootstrap (blue shaded area). Testing for differences in average power-envelope correlations revealed significant differences [T_1_ < 0.05 uncorrected] at the following frequencies: 1.2Hz, 1.4Hz–1.7Hz, 3.7Hz–4Hz, 8Hz–9.5Hz, 52Hz–64Hz. **(c)** TFCE permutation F-test (two-tailed) on average power-envelope correlations pointed at a non-significant cluster between 8.3Hz and 8.9Hz [T_2_: p < 0.25] for AD progression versus stable MCI. **(d)** Multivariate detection of group differences in power-envelope correlation matrices between using Riemannian-distance MANOVA. The black line represents pseudo-F scores [T_4_], the orange line the p-value [T_4_] corrected using the False Discovery Rate (FDR). The shaded green region indicates frequencies with T_4_: p < 0.03. The analysis revealed significant differences in power envelope metrics across frequencies ranging from 2.3Hz to 64Hz, suggesting wide-ranging differences in the power-envelope correlation architecture across frequencies **(e)** Topographical maps of power envelope correlation differences between groups, shown at the five frequencies listed in panel (b). AD progression showed a pattern of decreased power envelope in alpha frequency-range in posterior regions, which did not reach statistical significance.

**Fig. 4:**
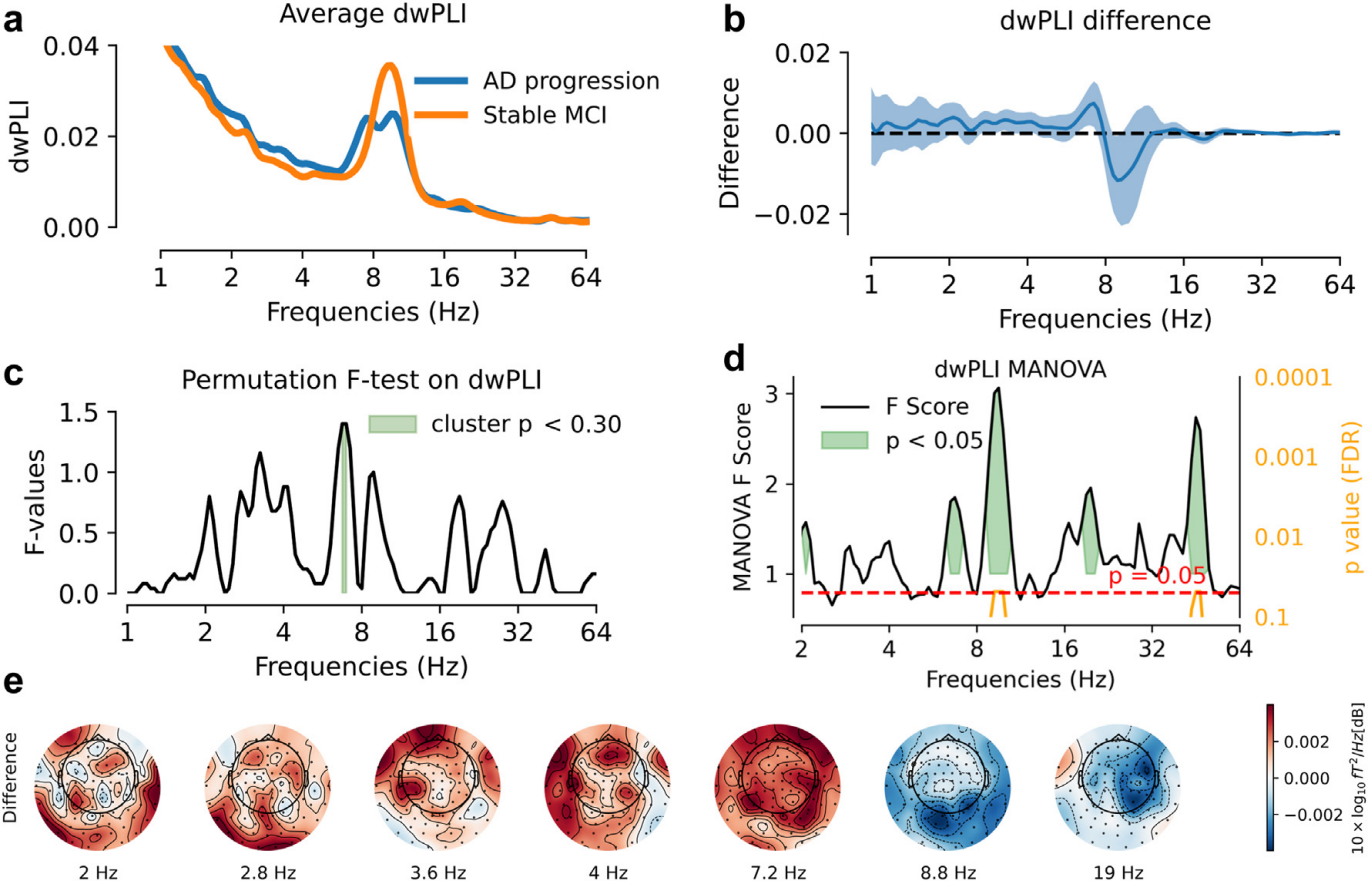
**AD-progression showed narrow-band alteration of phase interactions.** Analysis was based on all n = 117 cases. **(a)** Average dwPLI over all sensors: AD progression was visually associated with reduced dwPLI around 8–10Hz. **(b)** dwPLI difference between groups (blue line) and 95% confidence interval computed by bootstrap (blue shaded area). Testing for differences in average power-envelope correlations revealed significant differences [T_1_ < 0.05 uncorrected] at the following frequencies: 2Hz, 2.7Hz, 3.1–3.5Hz, 3.9–4.1Hz, 6.3–7.2Hz, 8.6–9.2Hz, 19–19.7Hz, 27.9Hz. **(c)** TFCE permutation F-test (two-tailed) on average power-envelope correlations pointed at a non-significant cluster between 6.7Hz and 7Hz [T_2_: p < 0.25]. (**d)** Multivariate detection of group differences in dwPLI matrices between using Riemannian-distance MANOVA. The black line represents pseudo-F scores [T_4_], the orange line the p-value [T_4_] corrected using the False Discovery Rate (FDR). The shaded green regions indicate frequencies with T_4_: p < 0.05. Significant differences were identified at the following frequencies: 2.1Hz, 6.5–7Hz, 8.9–10.2Hz, 19–20.4Hz, and 43.7–48.5Hz. After FDR correction, significant differences remained for frequencies between 9.2–9.8Hz and 45.3–46.9Hz (orange indicators). **(e)** Topographical maps of dwPLI differences between groups, shown at the seven frequencies listed in panel (b). In posterior brain regions, AD progression showed a pattern of decreased dwPLI in alpha band and increased dwPLI in theta band (not statistically significant).

We next explored the presence of informative differences in measures associated with long-range neural interactions beyond the power spectrum. Visual inspection suggested that AD progression was associated with reduced power envelope correlation (Fig. 3a and b) and reduced dwPLI around 8Hz (Fig. 4a and b). Results were not statistically significant following permutation F-test [T_2_] with multiple-comparison correction across all frequencies (Fig. 3c and 4c). However, Riemannian-distance MANOVA [T_4_] offered a different picture: for power-envelope correlations, we observed significant effects along the entire frequency spectrum (Fig. 3d); for dwPLI, we observed two significant frequency ranges, one above 8Hz and one above 40Hz (Fig. 4d). The first range was well aligned with the visual pattern revealed for the average dwPLI metric Fig. 4a and b. The second range was not anticipated by the average dwPLI metric, potentially offering novel information. In a descriptive topographical analysis, the AD progression group showed a spatially consistent pattern of decreased power envelope correlation and dwPLI in the alpha frequency band over posterior sensors but was not statistically consistent (Fig. 3e and 4e).

### Data-driven classification model of AD progression

**Fig. 5:**
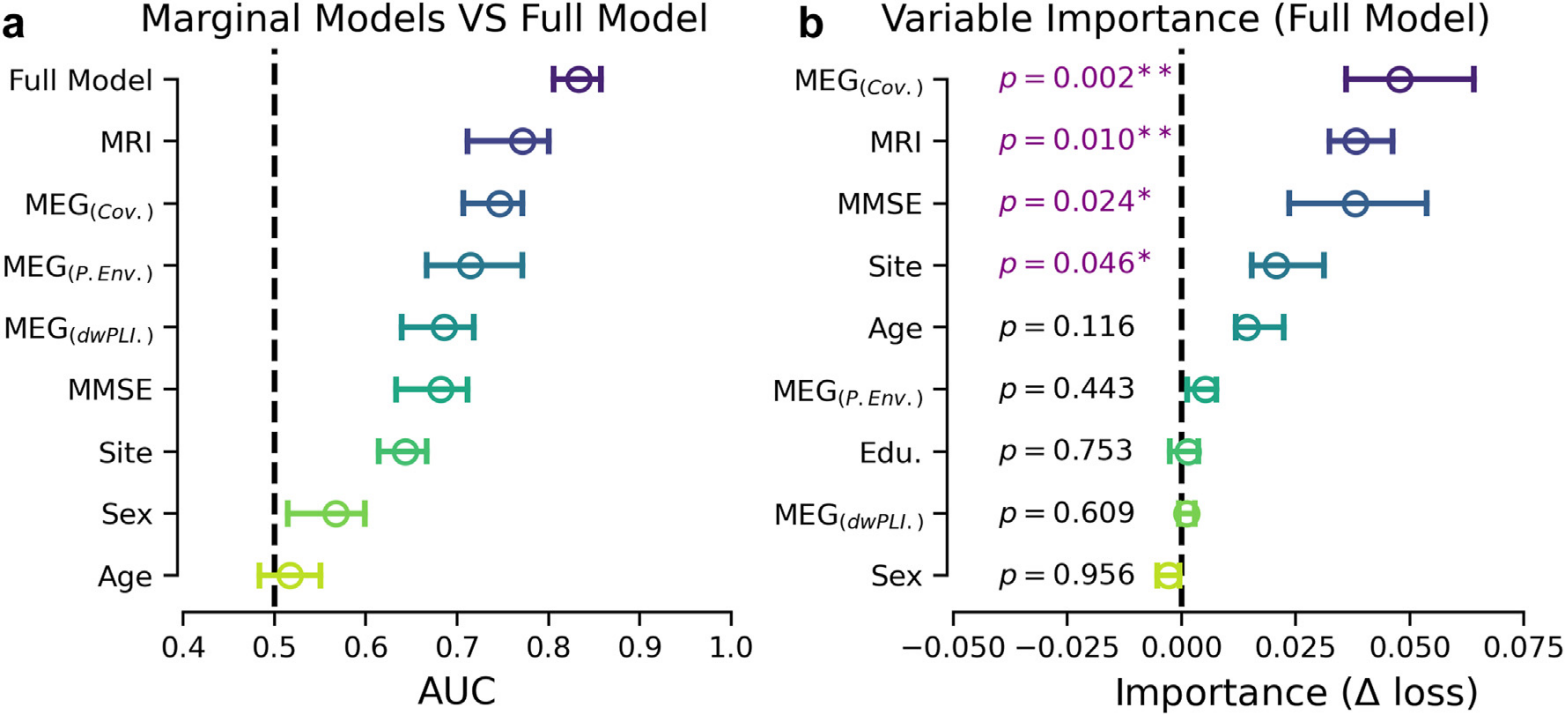
**Data-driven classification model of AD progression.** Analysis was based on all n = 117 cases. Panel (**a**) presents average cross-validated AUC scores, ordered by median scores. Error bars representing the 95% confidence intervals of the median over 100 cross-validation (CV) iterations. For marginal models using single demographic variables or the high-dimensional brain-data as inputs (MEG: Covariance, power envelope and dwPLI matrices along the frequency spectrum, MRI: Freesurfer cortical volumes alongside the full model combining all inputs using stacking (cf. Table 5 for details). One can see that brain-based models were ranked higher than single demographic predictors. The best result was obtained for the full model. Panel (**b**) presents average conditional permutation importance (CPI) scores capturing the change in the loss function upon removing the unique information given by a variable not shared with the other variables, ordered by median score. Error bars represent the 95% confidence intervals of the median over 100 cross-validation (CV) iterations. P-values referring to testing with pseudo t-statistic [T_5_] with Nadeau's and Bengio's corrected t-test. The results suggest that the performance of the full model was based on statistically complementary information from the MEG covariance, MMSE and MRI but also highlighted potential contributions from site effects.

To overcome the limitations of the previous analysis to a small number of pre-selected features, we explored constructing a data-driven classification model using machine-learning methods for combining high-dimensional multimodal data (Table 5, Fig. 5). This allowed us to multivariate modelling with all confounding variables and, including all MEG metrics at all sensors along the frequency spectrum as well as cortical volumes for all 64 regions of interest by the Desikan-Killiany parcellation.^76^ Marginal comparisons between stand-alone models using cross validation showed clear above-chance classification results for all models based on brain data but reconfirmed the importance of MMSE and site (Fig. 5a). MEG dwPLI was the weakest among the brain models (AUC = 0.67, SD = 0.15). MEG power envelopes showed improvements but higher variance (AUC = 0.71, SD = 0.15). The best marginal performance was observed for MEG covariances (AUC = 0.74, SD = 0.13) and MRI cortical volumes (AUC = 0.77, SD = 0.14). Combining all inputs led to markedly improved classification scores (AUC = 0.81, SD = 0.12), suggesting synergies between the different modalities. This raises the question of what inputs contributed independently to the full model. Analysis of conditional permutation importance (CPI)^93^, ^94^—accounting for shared information between variables—revealed that the full model relied on MEG covariances, MMSE, MRI and site (Fig. 5b). Importantly, age showed a visible impact on model performance although exceeding the nominal 5% significance level.

Taken together, these results add complementary validation to the findings from the previous statistical analysis with logistic regression (Fig. 2) by applying more conservative cross-validation rather than comparisons of in-sample discrimination and by avoiding selection of input variables after seeing the data. The results suggest this data-driven classification model could extract complementary information on AD progression based on MEG and MRI.

## Discussion

In this study, we investigated the potential of MEG as a tool for predicting the progression from MCI to AD dementia in the BioFIND dataset. We analysed data from 117 patients with MCI, among whom 64 eventually developed AD dementia (AD progression), while 53 remained cognitively stable (stable MCI). Continuous spectral analysis of the power spectrum with Morlet Wavelets enabled us to avoid bias from pre-specified frequency bands and to define a common signal representation for various spectral measures, i.e., power, covariance, phase interactions and power envelopes. Additionally, Riemannian methods allowed fine-grained multivariate analysis of covariance matrices while reducing signal distortion due to field spread. Our key findings revealed a significant reduction of MEG spectral power between 16Hz and 38Hz over parieto-occipital magnetometers in participants who later progressed to AD dementia. Moreover, covariance matrices analysed with Riemannian methods showed significant differences between groups, confirmed the differences in high frequencies (10–64Hz) and uncovered low frequency differences (<4Hz). Multivariate Riemannian-based MANOVA was also able to detect subtle alterations in neural interactions, measured by dwPLI and envelope-correlation. The posterior 16Hz–38Hz power reduction emerged as a robust predictor of future cognitive decline, even when considering conventional metrics like MMSE score and structural brain measures. Interestingly, adding MEG 16Hz–38Hz power improved logistic regression models based on hippocampal/total grey matter ratio. Notably, the data-driven classification model further demonstrated the complementary value of MEG and MRI. These findings highlight the potential of spectral power as a promising non-invasive electrophysiological biomarker to monitor AD progression, complementing classical diagnostic measures, including cognitive scores and structural MRI.

Patients with MCI who later progressed to AD dementia demonstrated reduced MEG power between 16 and 38Hz, in the beta band, localised to the parieto-occipital sensors. This frequency-specific effect is in line with studies showing decreased power in alpha and beta bands in participants with AD compared with healthy ageing, especially in the temporal and posterior/occipital brain regions.^21^^,^^23^^,^^30^^,^^31^^,^^47^^,^^98^^,^^99^ Claus et al. (1998)^100^ also reported that loss of beta band power was an independent predictor of an unfavourable prognosis in AD. Interestingly, a previous analysis of the BioFIND data^58^ found that sensor covariance in the low gamma range (30–48Hz) was most informative, but this was for the classification of MCI versus controls (orthogonal to the convertor/stable MCI distinction used here).

Three principal hypotheses have been proposed to explain the ‘slowing’ of brain activity in AD, e.g. increased low-frequency and decreased high-frequency activity. The main hypothesis is based on the cholinergic deficit, as correlation between loss of cholinergic neurons and increased delta and theta power has been shown in patients with AD.^47^ Moreover, the administration of cholinergic antagonists in animal and human models have shown to induce delta and theta activity.^101^ In our work, the AD progression group did not show increased delta nor theta power in the standard spectral analysis compared to the MCI stable group. However, our study revealed differences in the delta frequency band using Riemannian MANOVA, although without indicating the direction of these differences. A second hypothesis relies on AD being considered as a disconnection syndrome.^102^ Cortico-thalamic disconnection in particular could play a role not only in the increased delta activity, but also in the decreased beta activity observed in AD, and as observed here, as suggested by Holschneider & Leuchter (1995).^103^ A third hypothesis that could explain the slowing of brain activity in AD is the imbalance between excitatory and inhibitory neural activity. Altered excitatory-inhibitory dynamics have been shown to contribute to changes in oscillatory activity in AD. Modelling studies, such as those by Ranasinghe et al. (2022)^104^ and Verma et al. (2024),^105^ suggest that disruptions in these dynamics can lead to changes in power spectra, including both increased delta-theta power and decreased higher frequency activity. These findings indicate that abnormalities in the balance of excitation and inhibition in neural circuits could underlie the changes we observe in MEG power in AD progression. Further investigations would be needed to determine causative factors and provide a more comprehensive understanding of spectral power changes during AD progression.

An important finding is that covariance matrices analysed with Riemannian methods exhibited significant differences between groups across a wider range of frequencies than spectral power alone, particularly in the lower range of 1–4Hz, suggesting higher sensitivity of covariance versus power to identify future decliners. Riemannian tools have been shown to reduce signal distortion and are robust to field spread, potentially bypassing the need for source localisation with a biophysical model^56^^,^^106^ while implying a logarithmic function representing log power. These tools enabled us to capture signal changes in lower frequencies that were not consistently detectable based on pure sensor space power, which is systematically distorted by MEG field spread or EEG volume conduction. Importantly, prior work on age-prediction showed that the Riemannian embedding improved prediction performance compared to log power in sensor space,^56^ reaching equivalence with source power analysis. However, adding Riemannian embeddings after source analysis did not improve performance.^56^ We therefore propose our Riemannian signal as a potential surrogate for source power analysis, potentially facilitating the search of diagnostic and prognostic biomarkers for neurodegenerative diseases. We, therefore, recommend them as features for future statistical modelling and machine learning analyses.

Our results highlight the strength of multivariate Riemannian-based MANOVA in detecting subtle alterations in neural interactions, measured by dwPLI and envelope-correlation. This approach captured significant group differences across a broad frequency range for power envelope correlations and in specific alpha and high-frequency bands for dwPLI, showcasing its superior sensitivity compared to univariate analyses. Interestingly, the observation that power envelopes are broadly modulated may suggest underlying cross-frequency coupling mechanisms, as proposed by Canolty and colleagues,^107^ where the interaction between slow oscillations and faster rhythms could play a role in neural network dynamics. In contrast, traditional averaging methods were less effective, with visual inspection indicating reduced power envelope correlation and dwPLI in the alpha band for AD progression, findings that did not survive corrections for multiple comparisons. These results underscore the importance of multivariate approaches in revealing fine alterations in neural dynamics that might otherwise be overlooked.

Importantly, the data-driven classification model performed effectively when based solely on covariance features, suggesting potential redundancy between covariance, dwPLI, and power envelope metrics. However, that the model did not gain added information from dwPLI and power envelopes does not mean that these metrics do not, in principle, provide complementary information but could, rather, be due to the small size of the dataset. For example, in a brain-computer interface context, synergistic effects were reported for a related set of spectral metrics.^108^ Extracting additional benefit from those more subtle signals over covariance matrices may require larger datasets. Moreover, dwPLI and power envelopes can provide mechanistic insights into neural processes and functional architectures,^109^^,^^110^ making them valuable especially for experimental research. For small datasets, focusing on robust measures like power and covariance may be more practical for predictive modelling, given their efficiency and sensitivity.

Regarding our finding showing a trend towards decreased alpha coupling in AD progression, it contrasts with previous studies that have shown increased alpha synchronisation in posterior regions in MCI converters, interpreted as compensatory mechanisms or neurotoxicity of amyloid load.^46^^,^^111^ However, the spectral pattern we describe resembles the one usually found in patients with AD dementia, e.g. reduced synchronisation in alpha and beta frequency bands.^21^^,^^37^^,^^39^^,^^112^^,^^113^ One explanation could be that the patients with MCI in our study were already more advanced in the disease course, as suggested by a mean MMSE of 25.5 in the AD progression group in our study, compared to 27.4 and 27.7 in the study by López et al. (2014)^46^ and Bajo et al. (2012)^111^ respectively. This would be consistent with the proposal of Pusil et al. (2019),^114^ that discrepancies between previous MEG/EEG comparisons of MCI versus controls, relative to comparisons of MCI versus AD dementia, reflect the possibility that functional connectivity follows an inverted-U shape as a function of disease progression, with increased (hyper)connectivity from healthy controls to MCI, followed by decreased (hypo)connectivity from MCI to AD dementia. Only very few studies have compared functional connectivity metrics in progressive and stable patients with MCI,^36^^,^^46^^,^^111^ but this comparison should resemble more comparisons of MCI versus AD dementia (rather than healthy controls versus MCI), consistent at least with the decreases we found in coupling. However, this result should be interpreted with caution as we discuss subtle patterns of spatially averaged spectral metrics that did not reach statistical significance, unlike the MANOVA results which made better use of the spatial information to detect altered frequency ranges but did not provide topographic readouts.

Posterior MEG spectral power (16Hz–38Hz) showed stronger effects than conventional metrics as the MMSE score and structural brain measures in predicting progression from MCI to AD dementia. Combining age, education, MMSE, and posterior MEG power (16Hz–38Hz) led to better statistical explanation than combining age, education and MMSE with hippocampal volume/total grey matter in logistic regression models. The data-driven classification model demonstrated the potential of combining high-dimensional multimodal data for predicting AD progression, addressing limitations inherent in relying on a small number of pre-selected features. This approach incorporated MEG metrics across all sensors and frequencies, as well as cortical volumes from all 64 Desikan-Killiany^76^ regions of interest, allowing for a comprehensive analysis. Cross-validation revealed that models based on brain data achieved classification results above chance, with MEG covariances and MRI cortical volumes achieving the best marginal performance (AUC = 0.74, SD = 0.13 and AUC = 0.77, SD = 0.14, respectively). Notably, combining all inputs significantly enhanced classification scores (AUC = 0.81, SD = 0.12), underscoring the synergies between MEG, MRI, and clinical variables. The analysis of conditional permutation importance highlighted MEG covariances, MMSE, and MRI as key contributors to the full model's performance, with site effects also playing a role. These results provide complementary validation to findings from the logistic regression models by leveraging conservative cross-validation and avoiding biases introduced by pre-selecting features. The integration of MEG and MRI metrics in a data-driven framework highlights their potential to provide complementary insights into the mechanisms of AD progression and improve predictive accuracy.

The additive effect of MEG beta power and hippocampal volume/total grey matter ratio aligns with prior research suggesting selective associations between hippocampal volume and specific EEG frequency bands.^115^^,^^116^ Previous studies have demonstrated correlations between hippocampal volume and power in the alpha and theta bands, but not in the beta band.^37^^,^^117^, ^118^, ^119^ Thus, we would cautiously suggest that the observed beta power effect was statistically unrelated to hippocampal alterations.

The classification performances we obtained are in line with previous studies predicting conversion from MCI to AD dementia using EEG. Engedal et al. (2020)^32^ obtained effect sizes similar to those in our study with an AUC of 0.78, a sensitivity of 71%, a specificity of 69%, using a quantitative EEG Dementia Index and statistical pattern recognition method based on covariances. In our study, the fact that beta power emerged as a robust predictor of future cognitive decline is consistent with the study by Poil et al. (2013),^35^ which found that biomarkers sensitive to changes in the beta frequency (13–30Hz) band were the most optimal for predicting conversion from MCI to AD, after assessing 177 candidate EEG biomarkers. These authors hypothesised that AD progression is associated with less stable beta frequency, possibly related to a less efficient working memory, given that beta oscillations are believed to maintain the current sensorimotor, cognitive state and attention.^120^^,^^121^ Huang et al. (2000)^122^ found that patients with MCI who progressed to AD had a more anterior location of beta sources than stable MCI, and Baker et al. (2008)^123^ were able to classify MCI converters versus non-converters based on their EEG beta profile. On the other hand, increases in frontal beta power were observed in preclinical AD, and interpreted as compensatory mechanisms,^16^ suggesting a distinct mechanism, given the opposite direction of the effect and more anterior locus. Other EEG biomarkers found to be useful to predict decline from MCI to AD include decreased posterior alpha power,^23^^,^^122^^,^^124^ while Rossini et al. (2006)^36^ described higher power values in the delta, theta, and alpha 1 (8–10.5Hz) bands, mainly over temporal and parietal areas in converters.

There are fewer studies using MEG to assess the progression from MCI to AD.^47^ A resting-state MEG study conducted by Fernández et al. (2006)^45^ identified higher delta power in a left parietal region as a reliable indicator of conversion within a 2-year period. López et al. (2014)^46^ found an increase in phase synchronisation in the alpha band between the right anterior cingulate and temporo-occipital areas in AD converters. Our results are difficult to compare directly with these, because we only analysed power spectra in sensor-space, given our focus on more clinicallyapplicable MEG metrics (see Introduction).

A related point concerns the characteristic slowing phenotype that is hallmark of manifest AD. We did not observe a slowing in low frequencies, although the Riemannian covariance pointed at alterations in low frequencies. One reason might be that we compared only patients with MCI that may have already relatively progressed in the disease course as compared to healthy controls. To explore this point, we conducted an additional sensitivity analysis comparing the power spectra of healthy controls from the BioFind dataset versus patients with MCI (see Supplementary Figure S6 and Supplementary Figure S8). Our analysis revealed that patients with MCI exhibit increased delta-theta spectral power, which aligns with the expected cortical slowing described in the literature for the MCI stage. In another supplementary analysis comparing the power of AD progression group versus healthy controls (Supplementary Figure S7), AD progression also demonstrated increased delta-theta power in addition to a slowing of the alpha peak.

In our study, we achieved improved modelling results by integrating age, education, MEG power, and a simple volumetric MRI metric (hippocampal ratio). However, stronger effects would be required for definitive diagnosis, particularly when it implies decisions related to specific AD treatment in patients. This is in line with the proposition by Rossini et al. (2022)^125^ regarding a “biomarker pyramid” framework for cognitive risk evaluation, which foresees initial multimodal screening with widely accessible non-invasive methods (e.g. EEG, MRI, blood-based biomarkers, cognition) and follow-up or confirmation with expensive or invasive gold-standard approaches (PET, CSF). To realise this vision, it will be a crucial objective for future research to further decompose electrophysiological patterns and attribute its constituents to proteinopathies, structural and cellular anomalies as compared to cognitive function.

We shall first reflect on untapped potential for mapping cognitive function. While proteinopathies like amyloid-beta and phosphorylated tau are hallmark biomarkers for diagnosing AD, recent literature suggests they do not always effectively predict cognitive decline, especially in cognitively unimpaired people or at the MCI stage (e.g., Dubois et al., 2024).^126^ This highlights the importance of alternative biomarkers, including functional neurophysiological markers captured by MEG and EEG. Hyperexcitability and neurophysiological changes could indicate compensatory mechanisms or early dysfunction not captured by static measures like PET or CSF.^104^^,^^105^^,^^127^ Functional measures like MEG and EEG could provide insights into the balance between risk factors and protective factors in cognitive reserve, as discussed in recent papers such as Livingston et al., 2024 and Pappalettera et al., 2024.^128^^,^^129^ Indeed, EEG/MEG could help to: (1) summarise the risk related to metabolic and lifestyle factors, thereby acting as predictors of disease progression and cognitive reserve; and (2) monitor efficacy of amyloid lowering therapies over time by providing a physiological measure sitting in between amyloid biomarkers and clinical scales. This is important given the pressing issue of side effects such as amyloid related imaging anomalies (ARIA) and pseudo atrophy which, both, render measures of brain function desirable. In addition to monitoring treatment response, MEG and EEG biomarkers hold promise for personalising intervention strategies at the MCI stage. By leveraging an individual's unique neurophysiological profile, these tools could help predict cognitive prognosis and guide targeted interventions. This includes informing the need for specific therapies or lifestyle modifications that could help slow disease progression. Furthermore, such functional markers could provide physiological proxies for cognitive reserve, offering insights that are complementary to traditional biomarkers and clinical assessments. Extending these applications to pre-clinical stages of AD could further facilitate early intervention by identifying subtle neurophysiological changes before clinical symptoms emerge, ultimately supporting the development of preventive treatment strategies for individuals at risk.

To develop robust and specific brain-activity biomarkers that are useful for prognosis and treatment monitoring, it will be particularly important to better understand how different facets of M/EEG signals are associated with biomarkers of AD-specific proteinopathies, neurodegeneration, inflammation and synaptic function. i.e., a biomarker candidate should ideally be able to track processes beyond amyloid removal and instead capture recovery of brain function or changes in synaptic function. While this question regarding the interplay between proteinopathies and M/EEG activity is not new, the topic has received increasing attention in the recent literature.

For instance, Kudo et al. (2024),^130^ using event-based modelling on MEG signals, investigated neurophysiological trajectories in AD progression using MEG and found that alterations in neural synchrony in low frequencies, the alpha band and the beta band occurred progressively along the AD continuum, starting during the preclinical stage of the disease. Like in our study, a complementary role was observed for these brain-activity trajectories: these changes precede both neurodegeneration, as measured by grey matter atrophy, and cognitive decline. This suggests that MEG can detect functional changes related to amyloid pathology before clinical symptoms emerge.

Furthermore, using neural mass modelling on MEG signals, Ranasinghe et al. (2022),^104^ differentially attributed tau pathology and amyloid pathology to distinct brain-activity signatures in the low-frequency (2–7Hz), alpha-band (8–12Hz) and beta-band (13–35Hz) frequency ranges. The beta-band power modulation occupied a similar frequency range as our results, highlighting posterior parieto-occipital sources and showed a complex effect: increases in amyloid pathology measured with PET led to reduction of beta-band power whereas increases in tau pathology led to increases thereof. Applied to our findings this might suggest that the power-reduction signature we observed might imply differences in amyloid pathology.

Similarly, using MEG, Gallego-Rudolf et al. (2024)^131^ demonstrated a synergistic association of amyloid-β and tau pathology with cortical neurophysiology in AD, highlighting increases in delta-theta power (2–7Hz) and reduction of power in alpha band (8–12Hz) and beta band (15–29Hz) in patients with greater levels of amyloid and tau burden. Further regional analyses linked delta-band and alpha-band alterations with regional pathology changes in the entorhinal cortex, however, emphasising complex interaction effects that can lead to increases versus decreases in high-frequency activity depending on whether amyloid and tau pathology co-occurred.

For the greater ambition of contextualising M/EEG signatures of AD, the emergence of accurate CSF- and plasma-protein biomarkers provides an important opportunity. First of all, substituting e.g. phosphorylated tau (pTau-217, pTau-181) for costly neuroimaging results could add to our growing understanding of AD-specific pathophysiology.^9^ Second, the association between M/EEG signatures and unspecific neurodegeneration markers related to axonal damage, e.g. neurofilament light chain (NfL), could further help differentiate against co-pathologies.^132^^,^^133^ Furthermore, markers of neuroinflammation and synaptic function such as Glial Fibrillary Acidic Protein (GFAP) or Synaptosomal-Associated Protein (SNAP-25) could help contextualise M/EEG signatures in terms of underlying changes to neuronal function.^134^, ^135^, ^136^ Taken together this could provide clues for filtrating brain-activity patterns to achieve a more specific and interpretable description of brain function. Importantly, as neither plasma nor CSF data were available in the BioFind dataset, we could not directly link these MEG changes to specific biological markers.

To further differentiate and develop a specific role for electrophysiological brain-activity markers, it will be important to extend the use of EEG and/or MEG in clinical practice and research studies alongside protein biomarkers and imaging. Direct comparisons of EEG and MEG on the same participants would further support the goal of clinically validating MEG and translating it via EEG into a more widely accessible biomarker. In this regard, the recent AI-MIND initiative collecting electrophysiological (EEG, MEG) together with blood samples, genetics and imaging, holds promise to provide a relevant large data collection for further testing and developing the utility of M/EEG biomarkers for progression to AD.^137^ Such initiatives will therefore provide important opportunities for further validating and developing the findings from the current study and the recent literature which—while showing partial concord with our present findings—also pointed at potential nonlinear effects depending on the disease stage and the type of pathology.

While our study provides valuable insights into the potential of non-invasive electrophysiology as a predictive tool for the progression from MCI to AD dementia, there are certain limitations that should be considered. Even if the clinical diagnosis of AD dementia was done by neurologists in specialised memory clinics, the lack of CSF or amyloid PET biomarkers introduces a risk of misdiagnosis approximating 30%.^138^^,^^139^ Since the MEG recordings in our study were conducted between 2009 and 2016, the 2011 AD diagnostic criteria^60^ were the established and appropriate standards during that period, involving clinical and neuropsychological evaluation in addition to brain MRI but not biological biomarkers or amyloid/tau PET-scans. Incorporating CSF or PET biomarker assessments in future studies would refine the diagnostic specificity and enhance the reliability of the predictive models. Moreover, classification accuracy could be improved if additional data, such as APOE4 status or other biomarkers like blood-based biomarkers, become available in the BioFIND dataset in the future. Indeed, recent literature suggests that APOE4 may influence EEG patterns, potentially helping in differentiating individuals at higher risk for AD.^140^^,^^141^

It could have been worthwhile to explore the impact of signal duration used for estimation of the various metrics we compared in this work. However, the variable recording length between participants represents a limitation of the BioFind dataset as longer and equally long recordings would have provided an opportunity to systematically investigate the impact of the signal duration on the quality of MEG metrics. Although we only used the first 2 min of eyes-closed MEG data to minimise variability and reduce the risk of drowsiness, previous work has demonstrated that a duration of 2 min or even less was sufficient to estimate subjects’ specific patterns of brain activity. Wiesman et al. (2022) showed that 2 min of resting-state MEG recording were sufficient for reliable power metrics.^142^ Moreover, da Silva Castanheira et al. (2021)^143^ found that 30 s of MEG recording can be used for individual differentiation, and Paillard et al. (2025)^74^ demonstrated that even 10 × 10-s segments were effective across multiple prediction tasks. Additionally, we employed regularisation techniques to reduce variability and obtain stable estimates for covariance and interaction metrics (dwPLI and power envelope correlation). While short recordings are sufficient to capture individual-specific neurophysiological patterns, they may have limitations in distinguishing between periodic and aperiodic components. We could see in our results from numerical failure of Riemannian analyses, where the signal duration was too short. This was only the case for power envelopes below 2Hz, as those describe low-frequency dynamics not accessible with 10-s windows. Taken together, we would argue that the positive results obtained from our cross-validated classification model implies that the signal duration used in our study was sufficient to estimate stable brain-activity features.

Our study's sample size is relatively large compared to other MEG or EEG studies on MCI progression (n = 117), however it is still limited for developing prediction models. In the comparison of socio-demographic characteristics, we observed missing data for both education (n = 15) and MMSE (n = 5). To ensure completeness of our exploratory analysis, we applied mean imputation, using the mean of each variable calculated over all available subjects. Despite the use of a straightforward mean imputation approach in which missing features are predicted from all other features, we believe that the socio-demographic comparisons presented are reliable and unlikely to be significantly impacted by this imputation. Additionally, a more rigorous imputation method was utilised in the larger analysis model to address the potential shortcomings of the mean imputation and improve robustness. In our study, patients showing AD progression presented lower MMSE scores at baseline compared to stable MCI, which raises the possibility that our results reflect a later stage of MCI at baseline, rather than solely capturing the difference between subsequent converters and non-converters. This highlights the importance of considering the temporal dynamics of disease progression in our interpretation, and of longitudinal MEG/EEG assessment.^144^ Another potential limitation is that, although MoCA is known to be more sensitive to early cognitive variability compared to MMSE, the BioFIND dataset did not include MoCA scores, which prevented us from using it in this study. We acknowledge this as a limitation. However, recent studies suggest that electrophysiological measures such as MEG and EEG provide distinct and complementary information to general cognitive assessments like MMSE or MoCA.^145^, ^146^, ^147^ This highlights the potential of electrophysiology to provide valuable insights that complement traditional cognitive tests and could serve as powerful tools for predicting cognitive decline. Finally, there is likely variability between patients in terms of follow-up frequency and duration, but unfortunately, this information is not currently available in the BioFIND dataset. While this variability may reduce classification performance, it is unlikely to systematically bias the results obtained. Importantly, by including site as a covariate in our analyses, we have effectively accounted for site-specific effects, including differences in follow-up length, which strengthens the robustness of our findings. We recognise the exploratory nature of this study and the limited available participant information as regards information on neuropsychological tests or medications. Despite these limitations, we believe our work adds value by highlighting the utility of the BioFIND dataset as a resource for AD research, offering guidance for other researchers, and contributing to the growing body of literature on AD progression.

Future studies should expand the scope of our work to characterise neurodegenerative diseases other than AD. Assessing these biomarkers in diseases like dementia with Lewy bodies (DLB), frontotemporal dementia (FTD), and Parkinson's disease could provide a comprehensive understanding of their specificity and generalisability. Investigating how protein deposition alters electrophysiological patterns may uncover distinct pathways in various neurodegenerative disorders. Finally, our findings should be validated in EEG studies, going towards clinical application, as EEG is more widely applicable and cost-effective than MEG. This would facilitate the development of novel screening strategies in large populations of individuals with MCI, aligning with the emergence of new disease-modifying treatments for AD.

## Contributors

All authors have read and approved the final version of the manuscript.

R.H., D.V., F.M., ME.L. and R.B. have accessed and verified the underlying data.

In alphabetical order.

**Conceptualisation**: D.E., S.G.

**Data curation**: D.V., R.H.

**Formal analysis**: D.E., S.G.

**Investigation**: S.G.

**Methodology**: D.E., J.F.H., P.G., S.G.

**Project administration**: C.P., D.E, S.G.

**Software**: D.E., J.F.H.

**Supervision**: C.P., D.E.

**Validation**: D.E.

**Visualisation**: D.E., S.G.

**Writing—original draft**: D.E., S.G.

**Writing—review and editing**: C.P., D.E., D.V., F.M., J.F.H., ME.L., P.G., R.B., R.H., S.G.

## Data sharing statement

The data utilised in this study are derived from the BIOFIND dataset, a resource for researchers with institutionally controlled access via the Dementia Platform UK (DPUK). DPUK provided data/sample/ participant access for this project: DOI Biofind (https://doi.org/10.48532/007000) through MRC grant ref “MR/L023784/2” (core funding). BioFind was supported by grants EU JNPD (MR/P502017/1) and MRC (SUAG/046 G101400). The data (MEG and MRI raw data, clinical data) can be accessed by submitting a project proposal to DPUK. For further details, follow the instructions on the DPUK website (https://portal.dementiasplatform.uk/).

## Code sharing

All analysis code used in this study can be found at https://github.com/singaub/meg-biofind-ad-progression-paper-code.

## Declaration of interests

C.P. received payment or honoraria for advisory board from Lilly, Roche and Eisai. C.P. also received payments for consultation on the advisory boards of Lilly, Roche, Eisai and Novo Nordisk. C.P. received support for attending meetings, travel from Lilly, Roche and Eisai. P.G., J.F.H. & D.E. are full-time employees of F. Hoffmann–La Roche Ltd. F.M. received a grant from the Ministry of Science Government of Spain (grant PID2021-122979OB-C21).
