# Supplementary material for: Exploring the neuromagnetic signatures of cognitive decline from mild cognitive impairment to Alzheimer's disease dementia
Source: eBioMedicine. 2025 Mar 27;114:105659. doi: 10.1016/j.ebiom.2025.105659 (PMC11995804; doi:10.1016/j.ebiom.2025.105659)
Supplement: Supplementary Figures and Table [file mmc1.pdf]

Table of contents

Sensitivity analysis: Comparison to healthy controls (page 1)
Supplementary Figure 1 (page 2)
Supplementary Figure 2 (page 3)
Supplementary Figure 3 (page 4)
Supplementary Figure 4 (page 5)
Supplementary Figure 5 (page 6)
Supplementary Figure 6 (page 7)
Supplementary Figure 7 (page 8)
Supplementary Figure 8 (page 9)
Supplementary Figure 9 (page 10)
Supplementary Figure 10 (page 11)
Supplementary Table 1 (page 12)

Sensitivity analysis: Comparison to healthy controls

We conducted a supplementary analysis (see **Supplementary Figure 6**) comparing the spectral power of patients with MCI (n=157) with that of healthy controls (n=166) from the BioFIND dataset. Our analysis revealed that MCI patients exhibit increased delta-theta spectral power, which aligns with the expected cortical slowing described in the literature for the MCI stage. Additionally, we observed increased gamma power compared to healthy controls.

We have conducted another supplementary analysis (see **Supplementary Figure 7**) comparing the spectral power of the AD-progression group (n=64) and healthy controls (n=166). Our analysis revealed that the AD progression group exhibited increased spectral power compared to healthy controls at frequencies ranging from 1 Hz to 7 Hz (suggesting an increase in delta-theta power in the AD progression group) and from 39 Hz to 64 Hz (showing an increase in gamma power in the AD progression group). For the AD progression group, one can see a similar pattern, again showing a peak in the difference spectrum below 8Hz. This time the average peaks of the absolute spectra from both groups are even visually separated.

In another supplementary analysis (see **Supplementary Figure 8**), we compared the spectral power of Stable MCI (n=53) and healthy controls (n=166). Stable MCI patients demonstrate increased spectral power in frequencies ranging from 21Hz to 64Hz, and do not present any increase in low frequency spectral power, as compared to healthy controls.

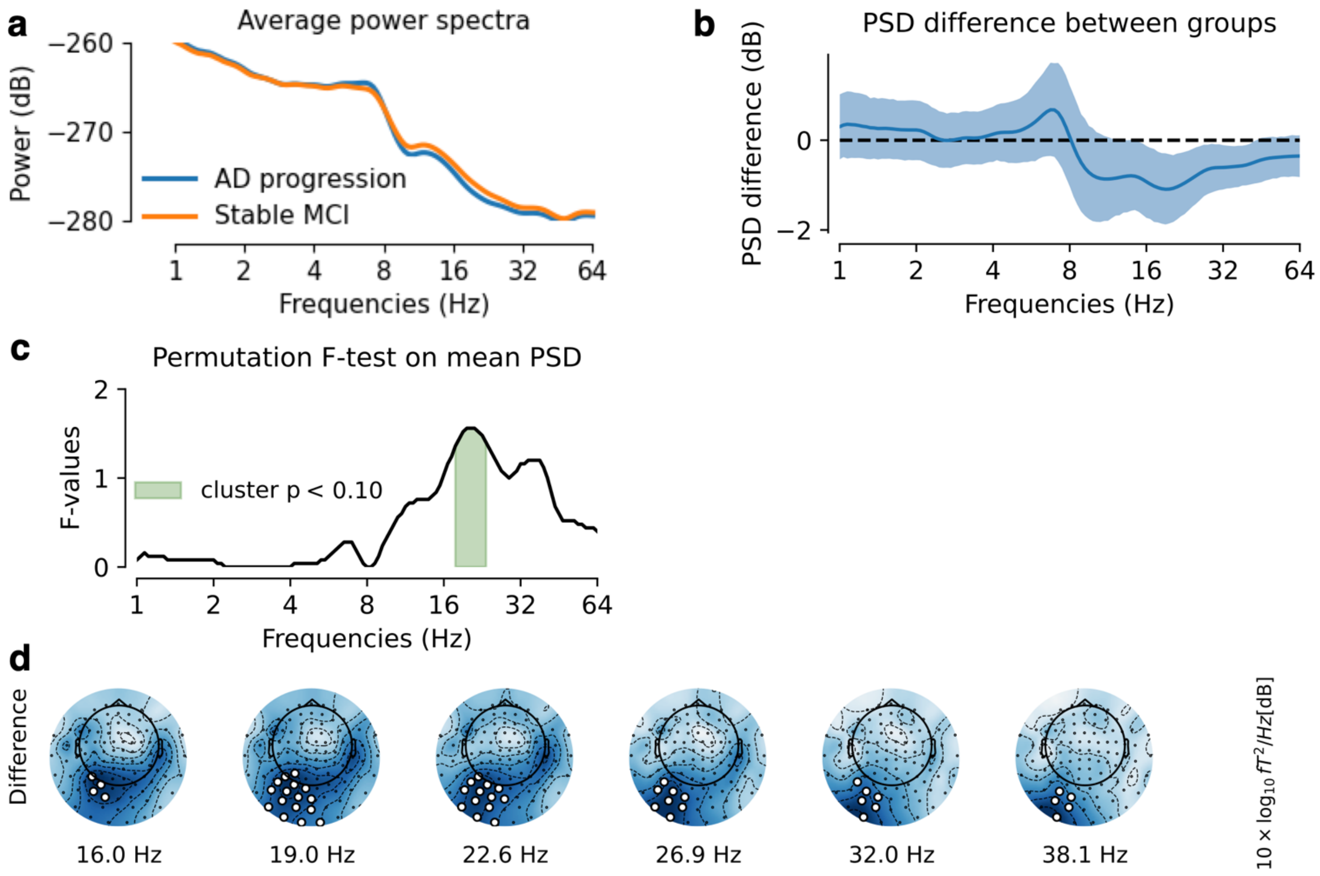

Supplementary Figure 1: AD progression shows reduced posterior 16 Hz to 38 Hz spectral power, adjusted for MMSE. Analysis was based on all n=117 cases. **(a)** Average power spectra over all sensors, adjusted on MMSE: upon visual inspection, AD progression showed a trend towards reduced spectral power between 15 Hz and 30 Hz. **(b)** Mean spectral power difference between groups (blue line) and 95% confidence interval computed by bootstrap (blue shaded area). **(c)** TFCE permutation F-test (two-tailed) on mean spectral power, adjusted on MMSE, showing a non-significant cluster between 17.8 Hz and 23.4 Hz [T_2: p<0.10] for AD progression versus stable MCI. **(d)** Topographical maps of spectral power difference between groups, adjusted on MMSE, showing reduced spectral power in left parieto-occipital region in AD progression group in frequencies from 16 Hz to 38 Hz. The white dots indicate significant differences [T3: p<0.05]. Only clusters that include three or more sensors are depicted. This result has been obtained by TFCE spatio-temporal cluster permutation test (two-tailed).

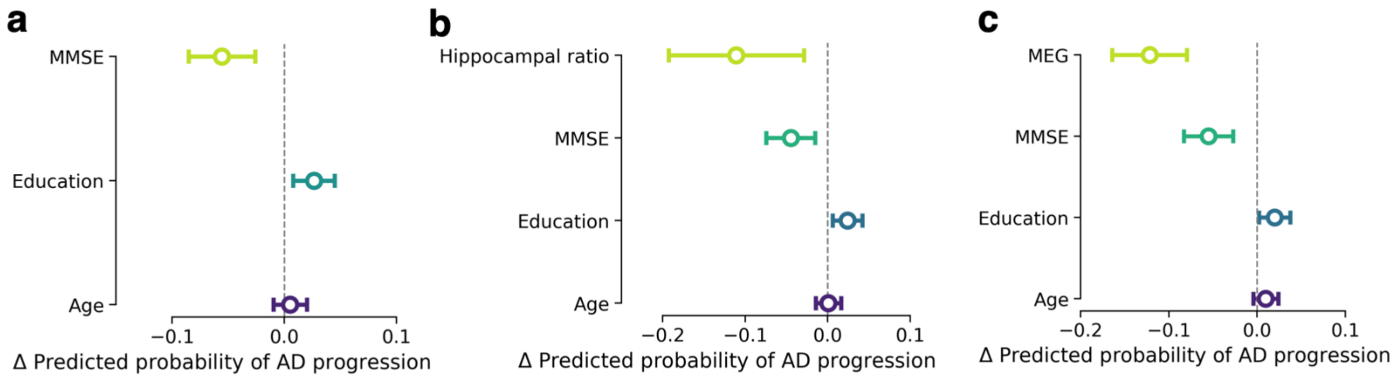

Supplementary Figure 2: Marginal effects display of logistic regression models of risk of progressing to AD dementia using different covariates. Analysis was based on the subset of n=104 containing both MEG and MRI. **(a)** Model 1 combines MMSE, education and age. Lower MMSE and higher education were significantly associated with a higher risk of progression to AD dementia conditional on all other variables. **(b)** Model 2 combines Hippocampus/Total grey matter ratio, MMSE, education and age. Lower hippocampal ratio, lower MMSE and higher education were significantly associated with a higher risk of progression to AD dementia, conditional on all other variables. **(c)** Model 3 combines MEG 16-38Hz spectral power in parieto-occipital regions, MMSE, education and age. Lower values of MEG 16-38Hz spectral power in left parieto-occipital region, lower MMSE and higher education were significantly associated with a higher risk of progression to AD dementia, conditional on all other variables.

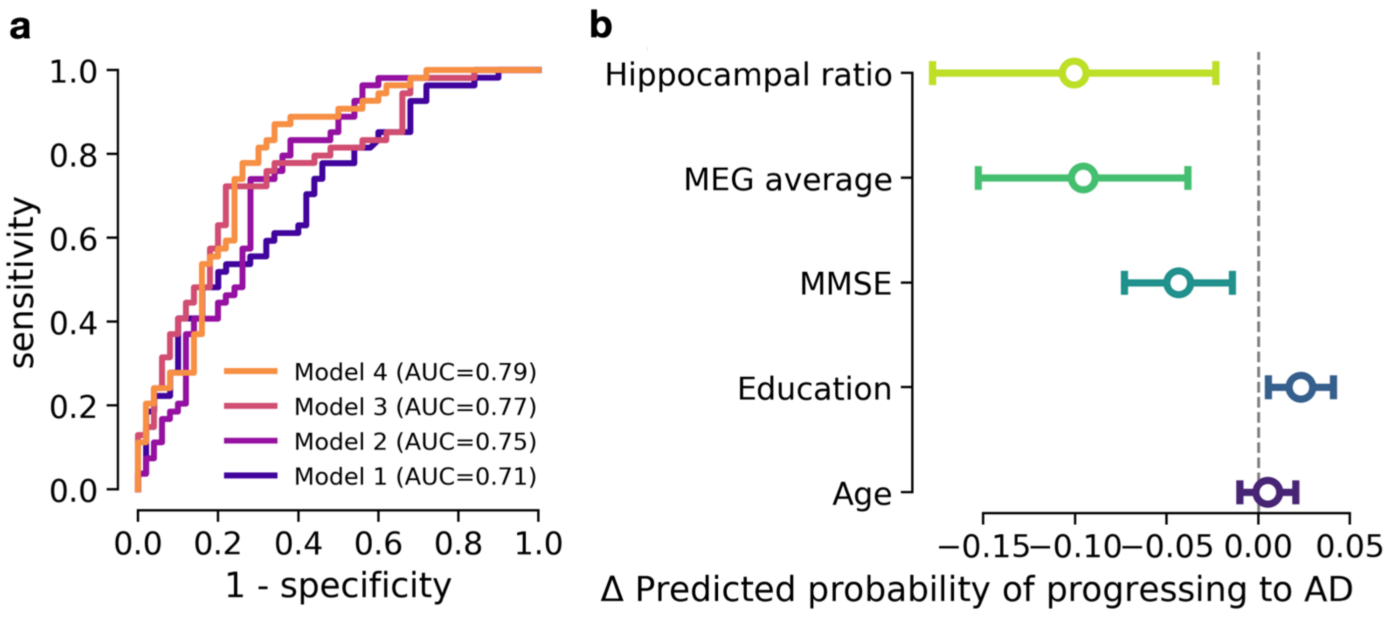

Supplementary Figure 3: Additive effect of MEG 16-38 Hz average spectral power over all sensors (adjusted for MMSE), hippocampal ratio and MMSE in logistic regression model. Analysis was based on the subset of n=104 containing both MEG and MRI. **(a) ROC curves of four logistic regression models to predict progression to AD dementia.** Model 1 combining age, education and MMSE had a 0.71 AUC (65% sensitivity and 58% specificity). Model 2 combining age, education, MMSE and Hippocampus/Total grey matter ratio had a 0.75 AUC (72% sensitivity and 72% specificity). Model 3 combining age, education, MMSE and average MEG 16-38 Hz power over all sensors had a 0.77 AUC (72% sensitivity and 72% specificity). Model 4 combining age, education, MMSE, average MEG 16-38 Hz power and Hippocampus/Total grey matter ratio achieved a 0.79 AUC (74% sensitivity and 74% specificity). **(b) Marginal effects display of logistic regression model of risk of progressing to AD dementia using the following covariates:** Hippocampus/Total grey matter ratio, MEG 16-38Hz average spectral power over all sensors (adjusted for MMSE), MMSE, education and age on the probability of progression to AD dementia. Higher values of MEG 16-38Hz average spectral power over all sensors, higher Hippocampus/Total grey matter ratio, higher MMSE and lower education were significantly associated with a reduced risk of progression to AD dementia conditional on all other variables.

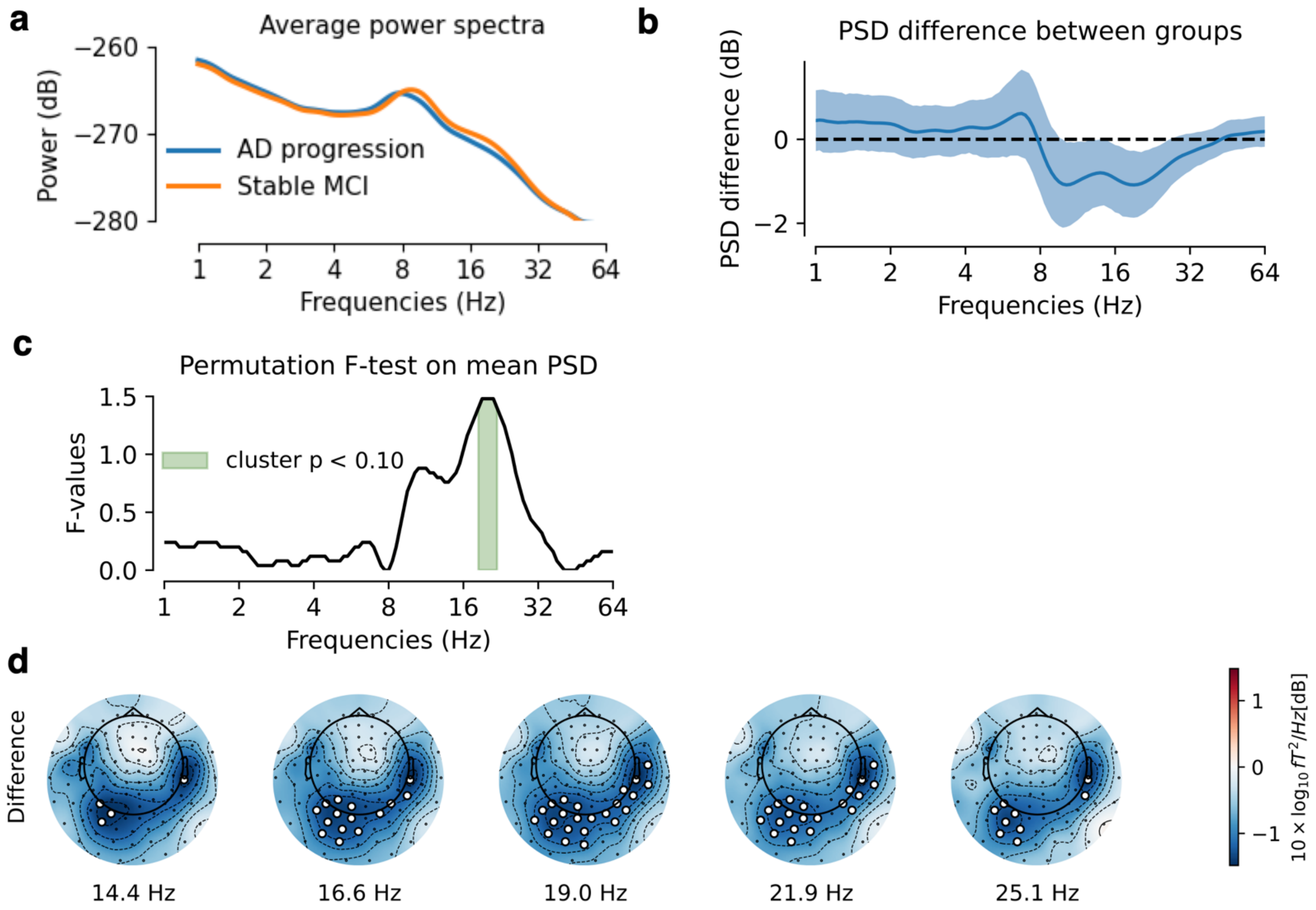

Supplementary Figure 4: Site-adjusted analysis reveals a trend towards decreased spectral power in beta band in posterior regions in AD progression group. Analysis was based on all n=117 cases. **(a)** Power spectra averaged over all sensors, adjusted for site effect: upon visual inspection, AD progression showed a trend towards reduced spectral power in alpha-beta frequency bands as compared to stable MCI. **(b)** Mean spectral power difference between groups (blue line) and 95% confidence interval computed by bootstrap (blue shaded area). The non-parametric two-tailed permutation test showed a p-value below 0.05 (uncorrected) for average spectral power for the following frequency range: from 10.2 Hz to 26 Hz. **(c)** TFCE permutation F-test (two-tailed) on mean spectral power, adjusted for site effects, showing a non-significant cluster between 18.4 Hz and 21.9 Hz [T_2: p<0.10] for AD progression versus stable MCI. **(d)** Topographical maps of spectral power differences between groups, using a p-value threshold of [T3: p<0.10]. AD progression showed a pattern of decreased spectral power in the beta band in posterior regions, which did not reach statistical significance. However, when using a p-value threshold [T3: p<0.10], AD progression group showed decreased spectral power from 14.4Hz to 25Hz in the posterior regions.

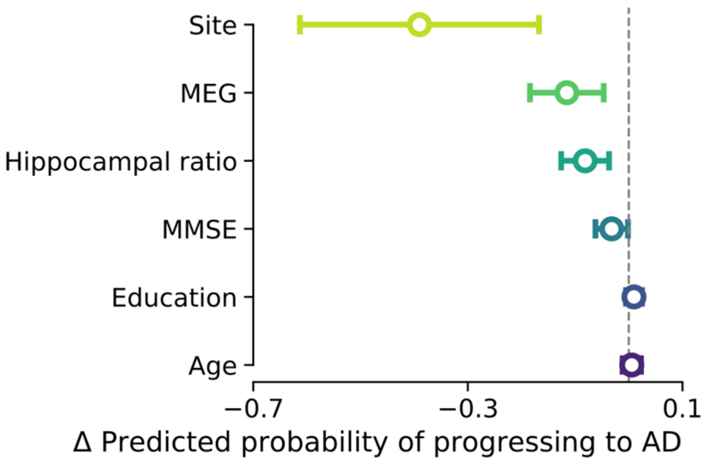

Supplementary Figure 5: Marginal effects display of logistic regression model of risk of progressing to AD dementia using the following covariates: Site (CTB/CBU), MEG 16-38Hz spectral power in parieto-occipital sensors, Hippocampus/Total grey matter ratio, MMSE, education and age. Analysis was based on the subset of n=104 containing both MEG and MRI. Higher values of MEG 16-38Hz spectral power in left parieto-occipital sensors, higher Hippocampus/Total grey matter ratio, higher MMSE and MEG recorded at site CTB were significantly associated with a reduced risk of progression to AD dementia conditional on all other variables. A higher level of education showed weaker effects in increasing the probability of progression to AD dementia.

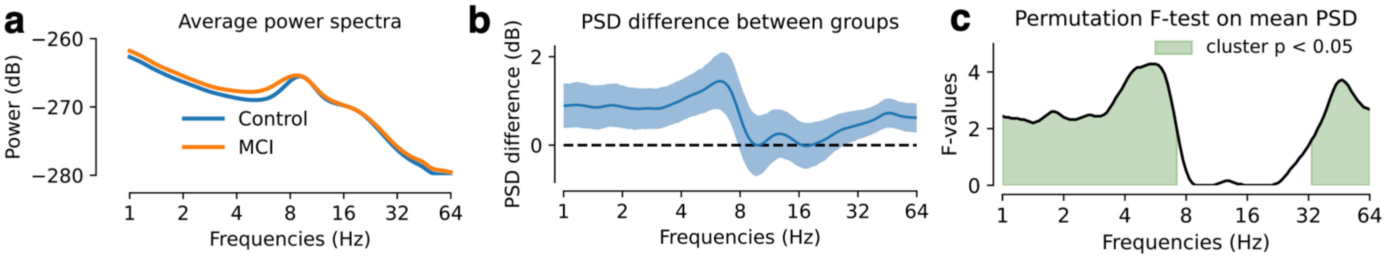

Supplementary Figure 6: Comparison of power spectra between patients with MCI (n=157) and healthy controls (n=166). **(a)** Average power spectra over all sensors: MCI patients demonstrate increased spectral power in frequencies ranging from 1Hz to 7Hz and from 33Hz to 64Hz, as compared to healthy controls. **(b)** Mean spectral power difference between groups (blue line) and 95% confidence interval computed by bootstrap (blue shaded area). **(c)** TFCE permutation F-test on mean spectral power, showing significant power difference between MCI patients and healthy controls at frequencies ranging from 1Hz to 7.2Hz (increase in delta-theta power in MCI) and 33.1Hz to 64Hz (increase in gamma power in MCI) [T_2: p<0.05].

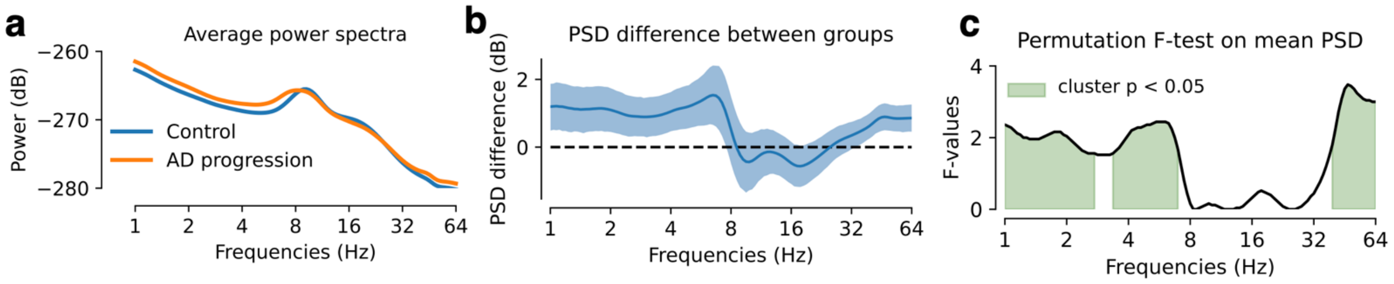

Supplementary Figure 7: Comparison of power spectra between AD-progression group (n=64) and healthy controls (n=166). **(a)** Average power spectra over all sensors: AD progression group demonstrate increased spectral power in frequencies ranging from 1Hz to 7Hz and from 39Hz to 64Hz, as compared to healthy controls. **(b)** Mean spectral power difference between groups (blue line) and 95% confidence interval computed by bootstrap (blue shaded area). **(c)** TFCE permutation F-test on mean spectral power, showing significant power difference between AD progression group and healthy controls at frequencies ranging from 1Hz to 7Hz (increase in delta-theta power in AD-progression group) and 39.4Hz to 64Hz (increase in gamma power in AD-progression group) [T_2: $p<0.05$].

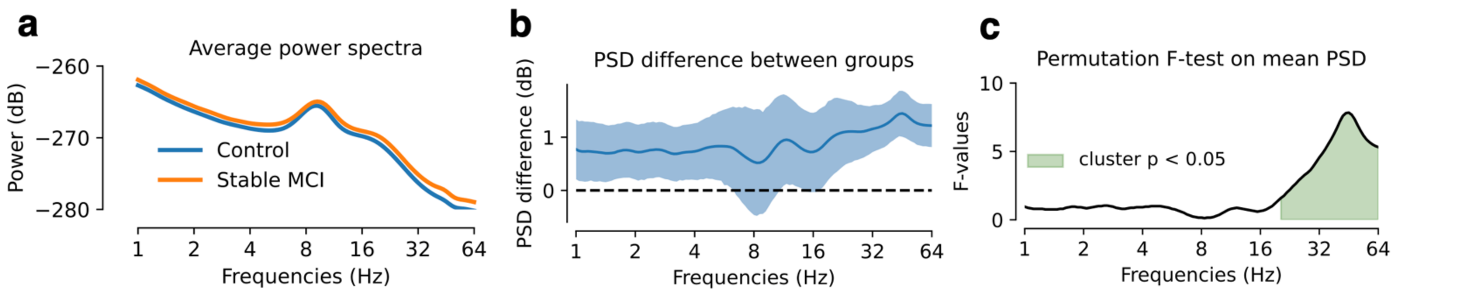

Supplementary Figure 8: Comparison of power spectra between Stable MCI (n=53) and healthy controls (n=166). **(a)** Average power spectra over all sensors: Stable MCI demonstrate increased spectral power in frequencies ranging from 21Hz to 64Hz, as compared to healthy controls. **(b)** Mean spectral power difference between groups (blue line) and 95% confidence interval computed by bootstrap (blue shaded area). **(c)** TFCE permutation F-test on mean spectral power, showing significant power difference between Stable MCI and healthy controls at frequencies ranging from 20.4Hz to 64Hz [T_2: $p<0.05$].

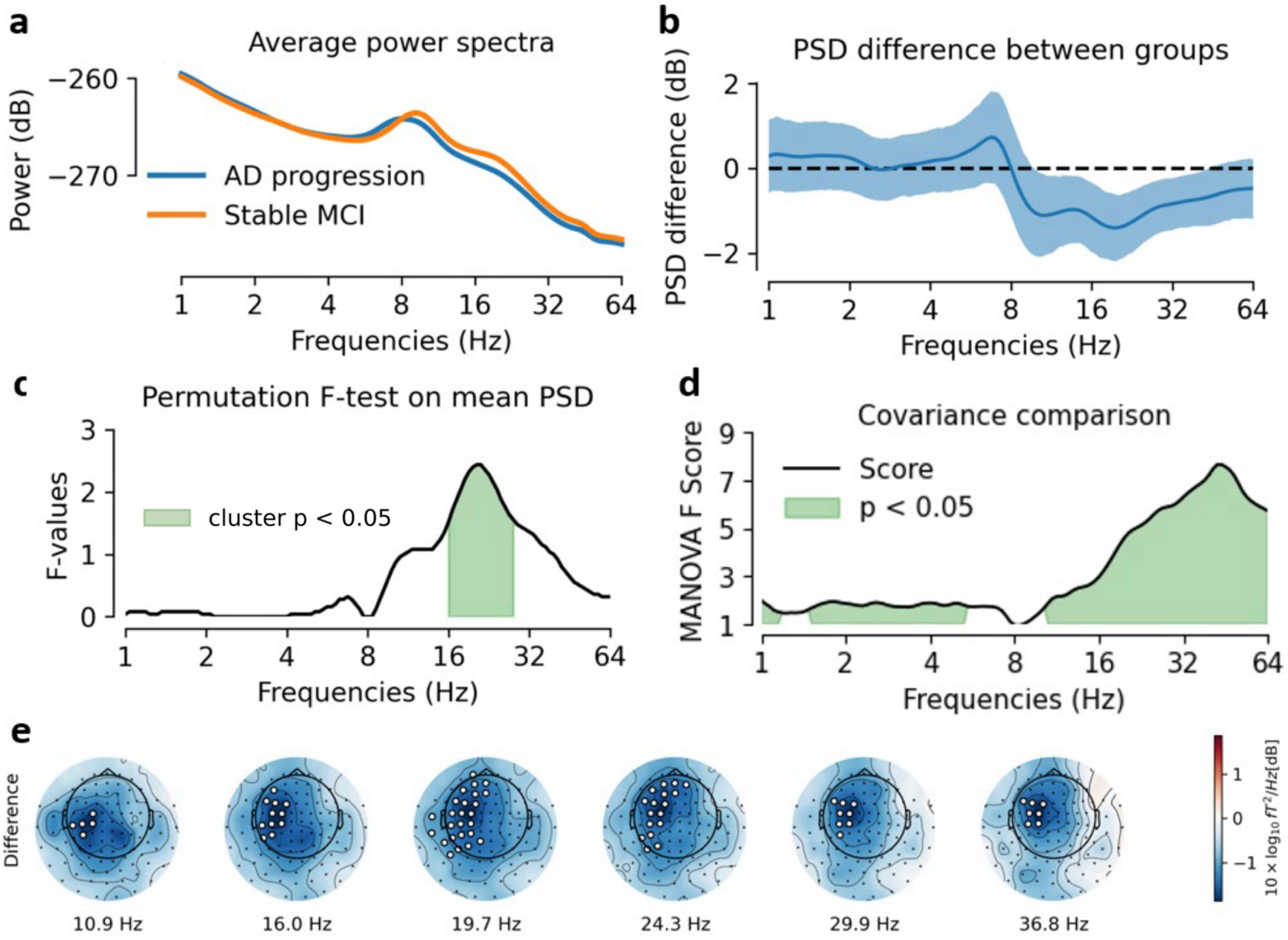

Supplementary Figure 9: Sensitivity analysis results showing effect of head alignment. Analysis was based on all n=117 cases. (**a**) Average power spectra over all sensors: upon visual inspection, AD progression was associated with reduced spectral power at baseline in frequencies ranging from 10 Hz to 40 Hz. (**b**) Mean spectral power difference between groups (blue line) and 95% confidence interval computed by bootstrap (blue shaded area). (**c**) TFCE permutation F-test (two-tailed) on mean spectral power, showing significant power difference between AD progression and stable MCI at frequencies ranging from 16 Hz to 27.9 Hz [T_2: p<0.05]. (**d**) Comparison between groups based on frequency-specific covariances using distance Manova, showing significant differences between 1 Hz to 6 Hz and 10 Hz to 64 Hz [T_4: p<0.05]. (**e**) Topographical maps of spectral power difference between groups, showing reduced spectral power in left fronto-temporo-parietal region in AD progression group in frequencies from 10.9 Hz to 36.8 Hz. The white dots indicate significant differences [T_3: p<0.05].

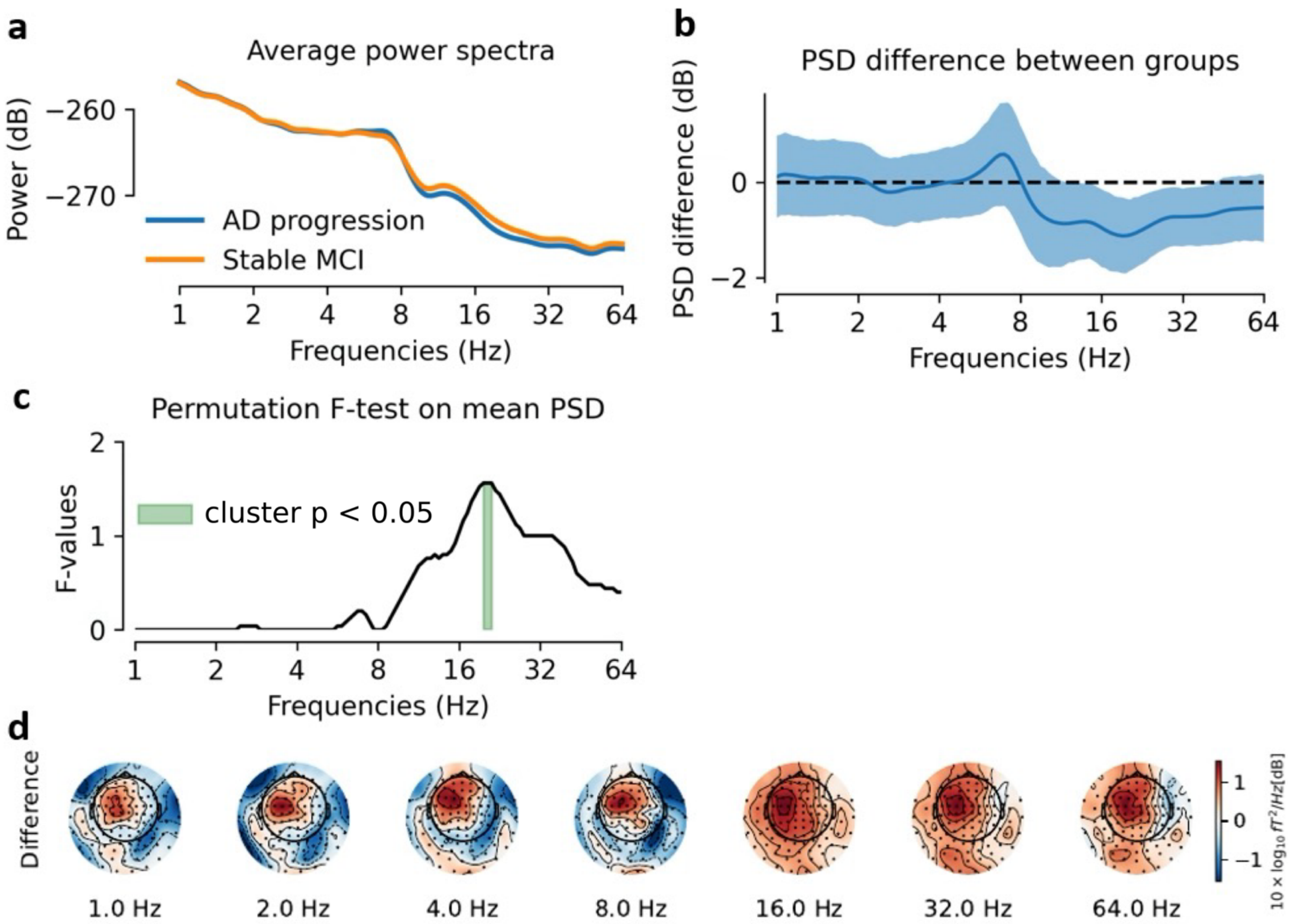

Supplementary Figure 10: Sensitivity analysis results showing effect of head alignment, after adjustment on MMSE. Analysis was based on all n=117 cases. **(a)** Average power spectra over all sensors, adjusted on MMSE: upon visual inspection, AD progression group demonstrated reduced spectral power in frequencies ranging from 15Hz to 36Hz. **(b)** Mean spectral power difference between groups (blue line) and 95% confidence interval computed by bootstrap (blue shaded area). **(c)** Permutation F-test (two-tailed) on mean spectral power, adjusted on MMSE, showing significant power difference between AD progression and stable MCI at frequencies ranging from 19.1 Hz to 21.1 Hz [T_2: $p<0.05$]. **(d)** Topographical maps of spectral power difference between groups, adjusted on MMSE, showing no significant differences in spatial patterns after adjusting on MMSE.

Factor	AME	SE	z	p	lower	upper
**Age**	0.007	0.007	0.944	0.345	-0.008	0.022
**Education**	0.020	0.009	2.30	0.022	0.003	0.038
**MEG 16-38Hz spectral power**	-0.060	0.016	-3.77	<0.001	-0.092	-0.029
**MMSE**	-0.036	0.015	-2.48	0.013	-0.064	-0.008
**MRI Hippocampal ratio**	-0.104	0.038	-2.71	0.007	-0.179	-0.029

Supplementary Table 1: Sensitivity analysis results showing effect of head alignment.
Marginal effects of Hippocampus/Total grey matter ratio, MMSE, MEG 16-38Hz spectral power cluster in fronto-temporo-parietal regions, education and age on the probability of progression to AD dementia. Analysis was based on the subset of n=104 containing both MEG and MRI. Higher values of MEG 16-38Hz spectral power in the left fronto-temporo-parietal region and higher Hippocampus/Total grey matter ratio were significantly associated with a reduced risk of progression to AD dementia. A higher level of education and lower MMSE were associated with an increased probability of progression to AD dementia. *AME = Average Marginal Effect; SE = Standard Error, z = z-value, p = p-value, Lower = Lower Confidence Interval, Upper = Upper Confidence Interval.*
